# A cell topography-based mechanism for ligand discrimination by the T cell receptor

**DOI:** 10.1073/pnas.1817255116

**Published:** 2019-06-20

**Authors:** Ricardo A. Fernandes, Kristina A. Ganzinger, Justin C. Tzou, Peter Jönsson, Steven F. Lee, Matthieu Palayret, Ana Mafalda Santos, Alexander R. Carr, Aleks Ponjavic, Veronica T. Chang, Charlotte Macleod, B. Christoffer Lagerholm, Alan E. Lindsay, Omer Dushek, Andreas Tilevik, Simon J. Davis, David Klenerman

**Affiliations:** ^a^Radcliffe Department of Medicine, John Radcliffe Hospital, University of Oxford, OX3 9DS Oxford, United Kingdom;; ^b^Medical Research Council Human Immunology Unit, John Radcliffe Hospital, University of Oxford, OX3 9DS Oxford, United Kingdom;; ^c^Department of Chemistry, University of Cambridge, CB2 1EW Cambridge, United Kingdom;; ^d^Department of Applied & Computational Mathematics & Statistics, University of Notre Dame, Notre Dame, IN 46556;; ^e^Mathematics Department, University of British Columbia, Vancouver, BC V6T 1Z2, Canada;; ^f^Sir William Dunn School of Pathology, University of Oxford, OX1 3RE Oxford, United Kingdom;; ^g^Wolfson Centre for Mathematical Biology, University of Oxford, OX1 3RE Oxford, United Kingdom;; ^h^School of Bioscience, University of Skövde, 541 28 Skövde, Sweden

**Keywords:** T cell receptor, receptor triggering, single-molecule imaging, microvilli, dwell time

## Abstract

The T cell receptor (TCR) initiates the elimination of pathogens and tumors by T cells. To avoid damage to the host, the receptor must be capable of discriminating between wild-type and mutated self and nonself peptide ligands presented by host cells. Exactly how the TCR does this is unknown. In resting T cells, the TCR is largely unphosphorylated due to the dominance of phosphatases over the kinases expressed at the cell surface. However, when agonist peptides are presented to the TCR by major histocompatibility complex proteins expressed by antigen-presenting cells (APCs), very fast receptor triggering, i.e., TCR phosphorylation, occurs. Recent work suggests that this depends on the local exclusion of the phosphatases from regions of contact of the T cells with the APCs. Here, we developed and tested a quantitative treatment of receptor triggering reliant only on TCR dwell time in phosphatase-depleted cell contacts constrained in area by cell topography. Using the model and experimentally derived parameters, we found that ligand discrimination likely depends crucially on individual contacts being ∼200 nm in radius, matching the dimensions of the surface protrusions used by T cells to interrogate their targets. The model not only correctly predicted the relative signaling potencies of known agonists and nonagonists but also achieved this in the absence of kinetic proofreading. Our work provides a simple, quantitative, and predictive molecular framework for understanding why TCR triggering is so selective and fast and reveals that, for some receptors, cell topography likely influences signaling outcomes.

## Significance

One approach to testing biological theories is to determine if they are predictive. We have developed a simple, theoretical treatment of T cell receptor (TCR) triggering that relies on just two physical principles: (*i*) the time TCRs spend in cell–cell contacts depleted of large tyrosine phosphatases and (*ii*) constraints on the size of these contacts imposed by cell topography. The theory not only distinguishes between agonistic and nonagonistic TCR ligands but predicts the relative signaling potencies of agonists with remarkable accuracy. These findings suggest that the theory captures the essential features of receptor triggering.

T cells play a central role in immunity. The triggering of T cell receptors (TCRs) expressed on the surfaces of all T cells, following their interaction with peptides complexed with major histocompatibility complex (pMHC) proteins on antigen-presenting cells (APCs), sets T cells on course to respond to pathogens and tumors (1). The TCR’s capacity to distinguish between different pMHC is referred to as ligand discrimination, a process that crucially underpins immunological “self/nonself” recognition and T cell development (2). Ineffective ligand discrimination often leads to immune deficiency or autoimmunity (3). Despite its central role in immunity, the biophysical basis of ligand discrimination by the TCR is unclear, and understanding it is increasingly becoming a matter of considerable urgency. Engineered immune cells expressing repurposed or artificial antigen receptors comprise a powerful new class of cancer therapeutics (4, 5). The severe off-target activity and extreme toxicity observed in some instances (6–8), however, is at least partly reflective of our poor grasp of the interplay between TCR binding kinetics, ligand density, and discriminatory signaling.

In addition to being highly selective, TCR signaling is extremely sensitive and fast: binding to a single agonist pMHC is sufficient to induce TCR signaling within seconds (9, 10). However, agonist peptides often comprise a very small fraction of all of the peptides presented as pMHC, raising the issue of how high sensitivity and discrimination are achieved simultaneously (11, 12). Several attempts have been made to explain ligand discrimination based on the TCR acting autonomously in ways analogous to G protein-coupled and growth factor receptors, with limited success. In such cases, TCR-induced signaling is assumed to rely exclusively on pMHC binding, and, in general, little consideration is given to extrinsic factors that might also influence signaling outcomes. Kinetic proofreading (KP)-based theories, which introduce multiple signaling steps to create delays that enhance signaling fidelity, succeed in explaining TCR discrimination in principle (13–15), but this comes at a cost, i.e., reduced sensitivity.

TCR triggering results in the tyrosine phosphorylation of its cytoplasmic immunoreceptor tyrosine-based activation motifs (ITAMs) by the kinase Lck, which unleashes a cascade of chemical reactions in the T cell, leading to transcriptional changes and T cell activation. In addition to ligand discrimination and sensitivity, a complete theory of T cell activation would have to account for a large number of related observations, such as peptide antagonism (16, 17), the synergistic signaling effects of self and nonself ligands (18), serial receptor engagement (19–21), and force-induced changes in TCR/pMHC stability (11, 22, 23), to name but a few. In addition, we have recently shown that TCR triggering is not strictly ligand dependent since it occurs when T cells form large contacts with non–ligand-presenting surfaces from which cluster of differentiation 45 (CD45) is at least partially excluded (24). Attempts have been made to generate models of T cell activation that incorporate the cell-biological underpinnings of many of these phenomena (25–27), but such models often have to rely on numerous assumptions, making it difficult to be certain of their accuracy (28). An alternative approach is to start with a simple model whose predictive ability can be tested, so that the extent to which it captures the essential features of receptor signaling can be determined.

Here, we developed and tested a quantitative treatment of TCR triggering relying on just two physical principles: (*i*) TCR “dwell time” in cell–cell contacts depleted of large tyrosine phosphatases and (*ii*) spatial constraints on contact size imposed by cell topography. The model suggested that restricting TCR engagement to small areas of contact would be essential for effective ligand discrimination, which could be achieved without KP. The model also predicted the relative potencies of well-characterized pMHC ligands with great accuracy, suggesting it captures the essential features of TCR triggering.

## Results

### A Signaling Theory Relying on TCR Dwell Time at Close Contacts.

The notion that TCR triggering might depend only on TCR dwell time at phosphatase-depleted regions of close contact between T cells and APCs is embodied in the kinetic-segregation (KS) model of TCR triggering (29). The KS model proposes that, at such contacts, the TCR remains accessible to active kinases but is protected from phosphatases that would otherwise reverse its phosphorylation, resulting in the phosphorylated state being sufficiently long-lived for downstream signaling to be initiated. In this context, cognate pMHC binding, which can slow or even halt TCR diffusion (30, 31), is expected to promote signaling simply by increasing the TCR’s dwell time inside the close contact, increasing the probability of receptor triggering. Depletion of the phosphatases is considered to be a passive process, driven by differences in the size of CD45 versus that of signaling and adhesive molecular complexes that form at the T cell/APC contact (24, 32–34).

**Fig. 1.**
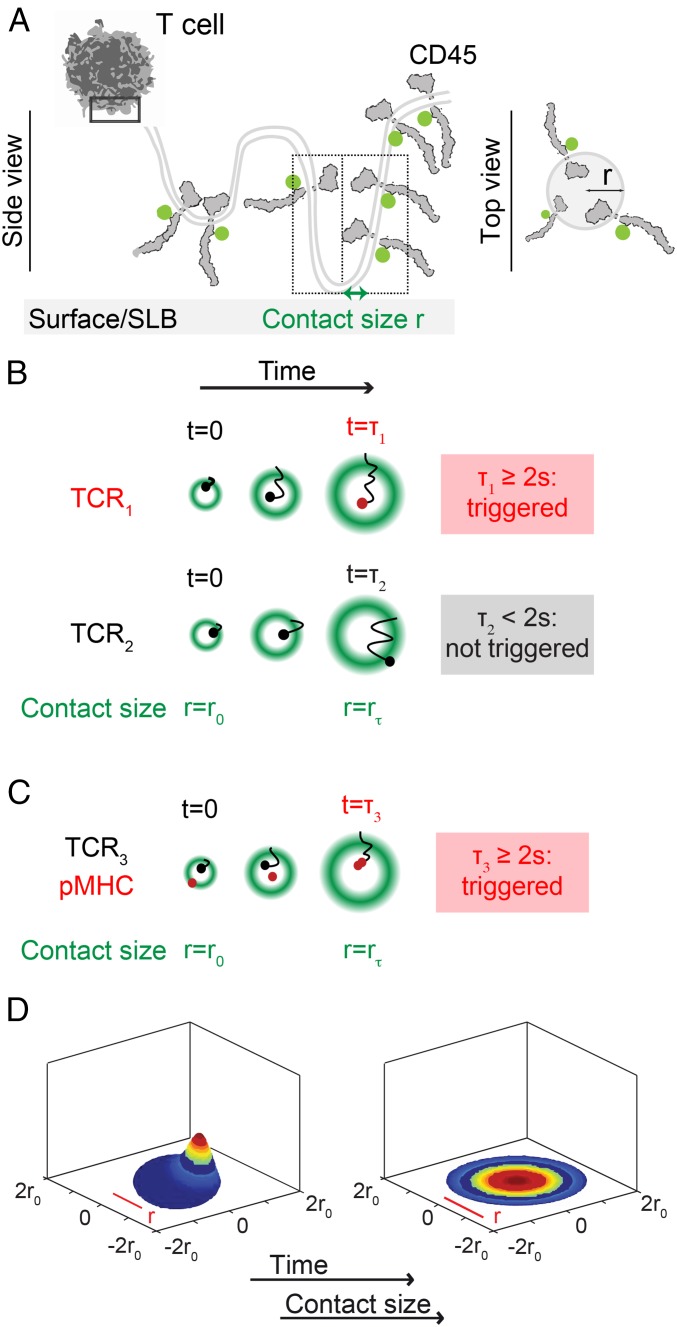
A quantitative treatment of TCR triggering relying on receptor dwell time at phosphatase-depleted close contacts. (*A*) Top and side views of the close contact depicting contact topography (with contact radius “r”) and CD45 exclusion. The first box (solid line) shows the region of the cell magnified below it. The second box (dotted line) shows the region depicted in the top view on the right. (*B*) According to the model, a TCR (TCR_1_) is triggered, i.e., phosphorylated because its residence time in the contact is ≥2 s. TCR_2_ is not triggered because it diffuses out of the contact in less than 2 s. (*C*) Also according to the model, a receptor (TCR_3_) that engages ligand is likely to be held in the contact ≥2 s and become triggered. In *B* and *C*, the margins of the contact are marked by the average positions of excluded CD45 molecules (green). (*D*) Snap shots from the simulation of the TCR density probability evolution in close contacts as they grow over time (*SI Appendix*, *Appendix I*).

Based on these ideas, we built a quantitative treatment of TCR triggering (Fig. 1; full details of the model are given in *SI Appendix*, *Appendix I*). We assumed (*i*) that when a T cell and an APC interact, “close contacts” are formed that each partially exclude CD45 (Fig. 1*A*), (*ii*) that TCRs diffuse in and out of the close contacts (Fig. 1*B*), (*iii*) that while the TCR is bound to a pMHC ligand it is unable to leave a close contact (Fig. 1*C*), and (*iv*) that any TCR that remains in a close contact for longer than a minimum time *t*_*min*_, irrespective of ligand binding, is “triggered,” i.e., a receptor ITAM is stably phosphorylated (Fig. 1 *B* and *C*). We took *t*_*min*_ to be 2 s, in line with observation (10, 26, 35–39) and in agreement with estimates of the catalytic activity of Lck [∼3 pTyr/s (40)] at the CD45/Lck ratio measured in contacts formed by T cells interacting with model surfaces (24). In this way, *t*_*min*_ creates an abrupt lower threshold for productive residence times. In addition to *t*_*min*_, the model incorporated the following parameters: (*i*) the rate of TCR entry into the close contact, (*ii*) the diffusion coefficients for unbound or ligand-bound receptors, and (*iii*) close-contact growth rate, thereby explicitly allowing for T cell topography and dynamics.

The model used a system of coupled partial differential equations (PDEs) to determine the distribution of TCR residence times, from which we could calculate the TCR-triggering probability, *p*, i.e., the likelihood that a receptor would have a dwell time >2 s, and therefore be phosphorylated. For freely diffusing TCRs in a circular close contact, the mean dwell time $\left(\tau_{TCR}\right)$ is dependent on contact radius, *r*, and the diffusion coefficient, *D*, of the receptor:

$$\tau_{TCR}=r^{2}/8D.$$

However, because close contacts are not static and instead increase in area over time (24, 41), we had to formulate and numerically solve PDEs with a moving-boundary condition to calculate the likelihood that the TCRs would remain in a close contact growing to radius r, assuming a circumference-dependent rate of TCR entry into the contact (the evolution of this probability distribution is shown in Fig. 1*D* and Movie S1; for further details, see *SI Appendix*, *Appendix I*). While multiple close contacts likely form between T cells and APCs, we modeled a single close contact only (triggering probabilities for multiple contacts can be obtained by multiplication, assuming the contacts are functionally independent). We used the model to ask the following questions: How can the TCR be triggered without ligands and how is this affected by close-contact area? Furthermore, what conditions would lead to robust discriminatory TCR triggering? Most importantly, using the known binding and signaling properties of well-characterized class I and II pMHC ligands, we tested whether the model was predictive.

### Parameterization of the Model.

To parameterize the model, it was necessary to determine the diffusional behavior of the TCR, Lck, and CD45 at close contacts. This was undertaken by studying the interactions of T cells with supported lipid bilayers (SLBs) with the defined membrane separation expected to be created in vivo by small adhesion molecules. For this, we used a signaling-disabled form of the rat adhesion protein CD48 (24). Jurkat T cells expressing CD48 (42) were allowed to settle onto SLBs presenting the extracellular domain of rat CD2 (rCD2), resulting in rCD2 accumulation and CD45 exclusion from the close contacts formed.

**Fig. 2.**
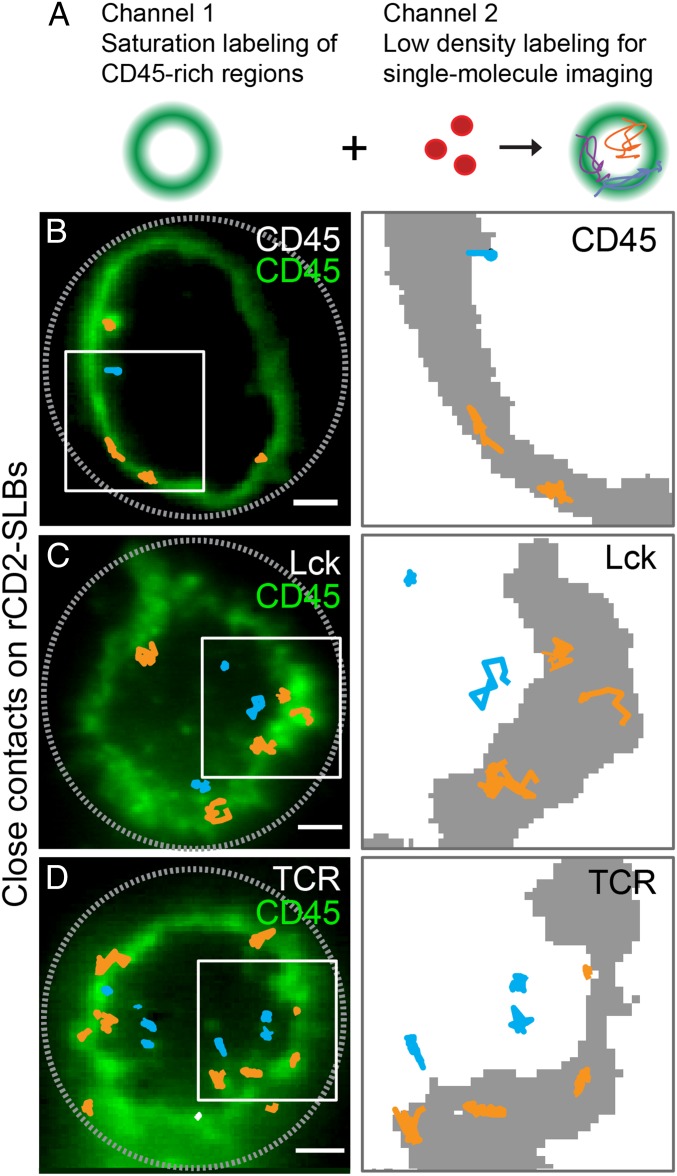
Parameterization of the model. (*A*) Experimental approach. High-density labeling of CD45 (Gap 8.3 Fab, Alexa Fluor 488) was used to indicate sites of close-contact formation between T cells and a rat CD2-presenting SLB (*Left*), and this was combined with simultaneous low-density labeling of CD45 (Gap 8.3 Fab, Alexa Fluor 568), Lck (Halo tag, tetramethylrhodamine [TMR]), or TCR (Halo tag, TMR) (*Right*) to enable TIRFM-based single-molecule tracking. (*B*–*D*, *Left*) TIRFM-based single-molecule tracking of CD45 (*B*), Lck (*C*), and TCR (*D*). Well-separated individual trajectories were recorded for >280 ms and colored according to position in the contact (orange in CD45-rich regions and blue in CD45-depleted regions). (*Right*) Close-up views of trajectories in regions marked by white rectangles; CD45-rich regions are shown in gray. (Scale bar, 2 µm.) Data are representative of three independent experiments with n > 10 cells.

Two-color total internal reflection fluorescence microscopy (TIRFM) and single-molecule tracking were used to follow substoichiometrically labeled TCR, Lck, or CD45 molecules relative to the boundaries of close contacts identified by CD45 bulk-labeled at high density in a second color (Fig. 2*A*). CD45 exhibited the most exclusion from rCD2-mediated T cell/SLB contacts. The density of CD45 molecules inside the close contacts was only 13 ± 3% of that outside (Fig. 2*B* and *SI Appendix*, Table S1), versus 56 ± 7% and 40 ± 6% for Lck and the TCR, respectively (Fig. 2 *C* and *D* and *SI Appendix*, Table S1). The initial CD45/Lck ratio of 5 to 1 before contact (24) was in this way reduced by ∼50% (*SI Appendix*, Figs. S1 and S2). Since it was not possible to measure the Lck/CD45 ratio at small, initial contacts, we obtained experimental values for larger, more stable contacts. However, bulk fluorescence measurements indicated that the CD45/Lck ratio did not vary significantly with contact growth: a CD45/Lck ratio of ∼2.7 was observed for all contacts of 1- to 2-μm radius (*SI Appendix*, Fig. S2). TCR diffusion rates were within the range reported by others (∼0.05 µm^2^/s; *SI Appendix*, Table S1 and Fig. S3; refs. 43–45). The effective catalytic activity of Lck at this CD45/Lck ratio has been shown to be approximately half-maximal (close to 2.2 pTyr/s; ref. 40). Mean diffusion coefficients for CD45, Lck, and the TCR were similar for molecules inside and outside the close contacts, and, overall, the TCR diffused ∼twofold more slowly than CD45 and Lck (*SI Appendix*, Table S1 and Fig. S3). Measurements used for the modeling that were made here or by others are summarized in Table 1 (a more detailed list of parameters is given in *SI Appendix*, Table S2).

**Table 1.** Experimental parameters used in this study

Parameters	Value
Total cell area	415 μm^2^*
TCR diffusion coefficient	0.05 μm^2^ s^−1^^†^
Number of TCRs per cell	41,500^‡^
Fraction of TCR segregation	0.62^†^
CD45 to Lck ratio	2.5:1^†^
Close contact radius	220 nm^§^
Half-life of T cell–APC contacts	120 s^§^
TCR triggering (minimum dwell time for triggering)	2 s^§^^,^^¶^

References are given for measurements taken from the literature.

* Weaver (65).

^†^ Experimentally determined in this study for Jurkat T cells.

^‡^ Experimentally determined in this study for CD4 T cells.

^§^ Cai et al. (49).

^¶^ Hui and Vale (40).

Two assumptions of the model that needed to be confirmed were (*i*) that CD45 is evenly distributed at the T cell surface before contact formation and (*ii*) that it is excluded as soon as close contacts begin to form. Three-dimensional superresolution imaging (46) showed that CD45 is indeed evenly distributed over the surface of the T cell, including the ends of microvilli (*SI Appendix*, Fig. S4*A*), consistent with previous findings (47). The early stages of close-contact formation are difficult to study on SLBs because the contacts grow quickly. T cells form close contacts with protein-coated glass much more slowly, however, and 2D superresolution imaging revealed that on this surface CD45 was excluded from contacts of ∼80 nm, smaller than the diffraction limit (*SI Appendix*, Fig. S4 *B* and *C*; see also ref. 41). Furthermore, when Jurkat T cells expressing CD48 interacted with SLBs loaded with fluorescently labeled forms of the extracellular domains of CD45RABC and rCD2, the SLB-bound CD45 was spontaneously excluded from contacts that formed (*SI Appendix*, Fig. S5 and Movie S2). These observations suggest that CD45 segregation occurs passively, that is, immediately upon contact formation, in line with previous findings (24).

### Validation of the Model.

**Fig. 3.**
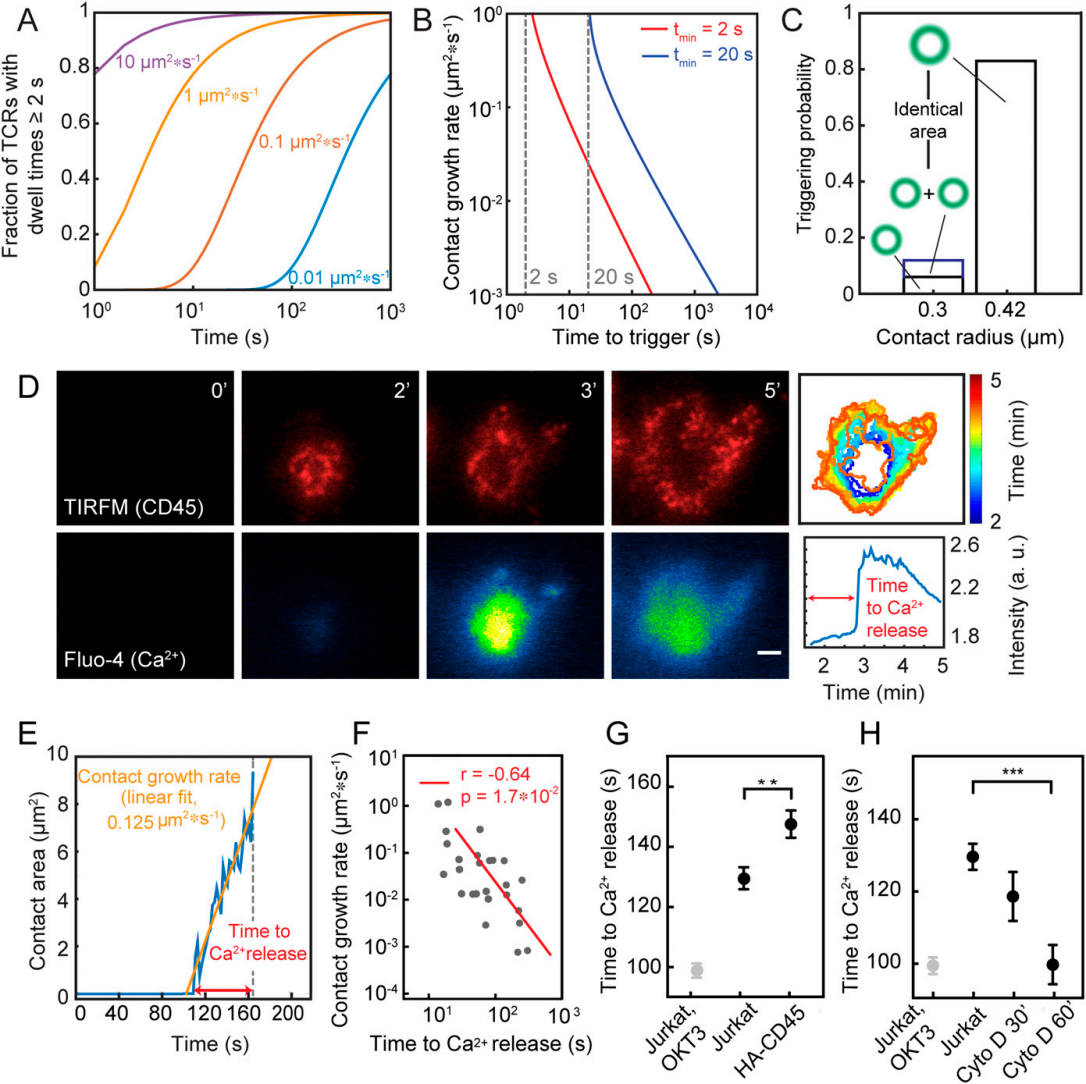
Experimental validation of the model. (*A*) Fraction of triggered TCRs as a function of time and contact growth rate (*t*_*min*_ = 2 s, D = 0.05 µm^2^/s, g = 0.01 to 10 µm^2^/s). (*B*) Time taken to TCR triggering as a function of close-contact growth rate. (*C*) Comparison of triggering probability for one versus two contacts or a single contact of double the contact area. (*D*) Dynamics of close-contact formation [CD45 fluorescence (Gap 8.3 Fab, Alexa Fluor 568), TIRFM] (*Top*) and Ca^2+^ release (detected as Fluo-4 fluorescence) (*Bottom*) for cells contacting rCD2-presenting SLBs. (Scale bar, 2 μm.) (*Top Right*) Color-coded representation of the temporal evolution of contact area over time. (*Bottom Right*) Temporal evolution of Fluo-4 intensity averaged over entire contact; n > 10 cells from five independent experiments. (*E*) Trace of a representative contact over time for growth-rate determination. (*F*) Relationship between close-contact growth rate and the time taken to triggering. (*G*) Time delay between initial contact of cells with rCD2-presenting SLBs and Ca^2+^ release for Jurkat T cells and cells expressing HA-CD45. (*H*) Time delay between initial contact of cells with IgG-coated glass and Ca^2+^ release in the presence of the actin depolymerizing drug cytochalasin D (data shown as mean time of calcium release for three independent experiments with >200 cells per condition; **P = 0.01 and ***P <0.001, two-tailed *t* test, unequal variance assumed; errors are SEM).

Our observation that the TCR can be triggered in the absence of ligands (24) supports our premise, i.e., that TCR triggering depends only on TCR dwell time in close contacts depleted of CD45. However, a number of testable predictions for signaling under these conditions allow experimental validation of the model. First, since TCR dwell time depends on close-contact size, which in turn is affected by close-contact growth rate (for contacts growing on similar time scales to TCR diffusion), triggering times ought to be shorter for cells with larger close-contact growth rates (*prediction 1*; Fig. 3 *A* and *B*; for further details see *SI Appendix*, *Appendix I*). Second, since the phosphorylation rate, i.e., the effective *k*_cat_ of Lck, is inversely proportional to the CD45/Lck ratio in the close contact, an increase in this ratio should lead to longer triggering times (*prediction 2*; Fig. 3*B*; for quantification of the effective Lck *k*_cat_ at different CD45/Lck ratios, see ref. 40). Finally, receptor triggering should occur sooner for single large contacts compared with two separate contacts of the same combined size (*prediction 3*). For example, the model predicts that the triggering probability would increase >sevenfold when two single contacts coalesce into a larger one (Fig. 3*C*).

We tested these predictions for CD48-expressing Jurkat T cells forming contacts with rCD2-presenting SLBs, using calcium release as a proxy for receptor triggering. To test *prediction 1*, we exploited the natural variation in close-contact growth rates. We simultaneously measured contact growth and signaling times by coupling TIRFM-based detection of close contacts, identified as regions of CD45 exclusion (24), with changes in calcium reporter fluorescence (Fluo-4; Fig. 3 *D* and *E* and Movie S3). In agreement with the model’s prediction, receptor triggering occurred faster for cells with larger close-contact growth rates (Fig. 3*F*). For testing *prediction 2*, we compared the triggering times for Jurkat T cells with those for cells expressing a form of CD45 lacking its extracellular domain (HA-CD45) (24). HA-CD45 is less efficiently excluded from contacts and therefore reduces Lck *k*_cat_ by increasing the CD45/Lck ratio in the close contacts (*SI Appendix*, Fig. S1; ref. 24). As predicted once again by the model, expression of HA-CD45 at ∼10,000 copies/cell (i.e., at 5% of total CD45 expression; *SI Appendix*, Fig. S6) delayed triggering by almost 20 s (∼15%, *P* < 0.05, two-tailed *t* test, unequal variance assumed; Fig. 3*G*). We previously showed, in the reverse experiment, that the forced exclusion of Lck from close contacts, i.e., by expressing the kinase as a chimera with the extracellular domain of CD45, also reduced the level of TCR triggering under these conditions (24). Finally, treatment of Jurkat T cells with cytochalasin D, an inhibitor of actin polymerization and microvillus formation (48), which produced larger and more stable contacts, reduced triggering times by up to 30 s (∼23%, *P* < 0.05) in a drug exposure-dependent manner, consistent with the third prediction of the model (Fig. 3*H*).

### Why TCRs Are Triggered in the Absence of Ligands.

**Fig. 4.**
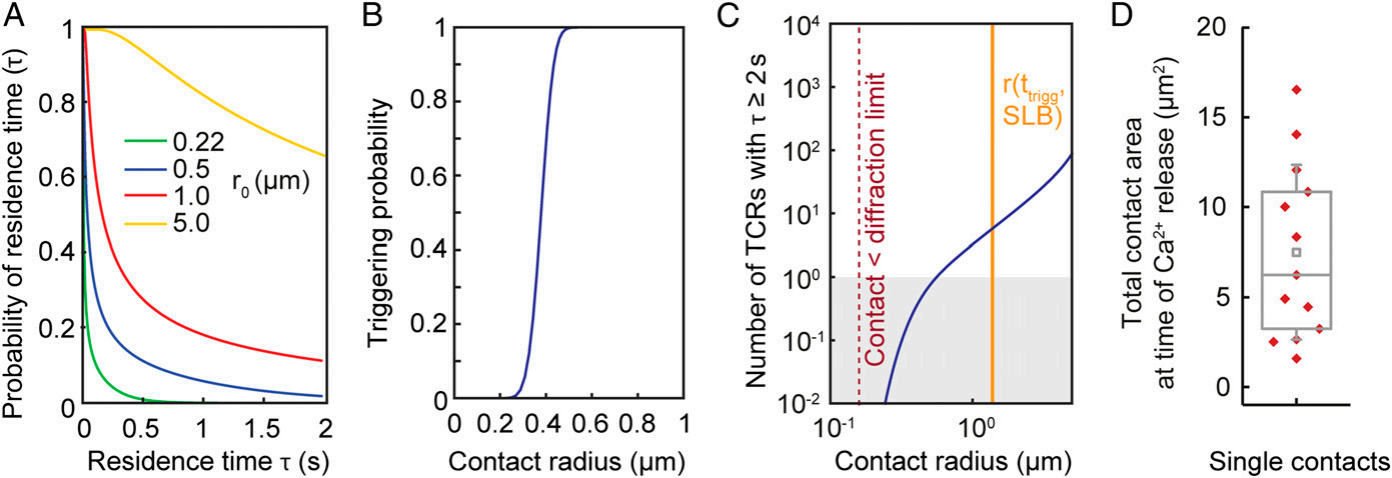
Why the TCR can be triggered in the absence of ligands. (*A*) Probability that a TCR remains inside a close contact for time, τ, for close contacts of varying fixed radius, r_0_. (*B*) Probability that a single TCR stays inside a close contact >2 s as a function of final close-contact radius for growing contacts. (*C*) Total number of TCRs that remain inside the close contact for >2 s, incorporating the estimates shown in *A*, the density of TCRs in Jurkat T cells, and the degree of exclusion of the TCR from close contacts for cells interacting with rCD2-presenting SLBs. (*D*) Total contact area (region of CD45 exclusion) at the time of calcium release for T cells interacting with rCD2-presenting SLBs (13 cells, 5 independent experiments). Central lines indicate the median; small squares indicate the mean; boxes show interquartile range; whiskers indicate SD.

Having validated the model, we first used it to explore the quantitative basis of TCR triggering in the absence of ligands. Our calculations showed that the probability of ligand-independent receptor triggering is highly sensitive to close-contact radius (Fig. 4 *A* and *B*). The probability, *p*, that the dwell time reaches *t*_*min*_ >2 s, is 0 for contacts of the size observed during T cell interrogation of APCs (220 nm; Fig. 4*A*; refs. 49 and 50), implying that no TCR is likely to be triggered in contacts of this size that lack ligands. On SLBs, however, T cells form contacts much larger than those observed during cell–cell interactions (Fig. 2 *B*–*D*), and for these types of contacts, we estimate that ∼16 TCRs will be triggered per contact in the absence of ligands (Fig. 4*C*). This calculation is based on (*i*) *p*, (*ii*) the total contact size observed at the time of calcium signaling (median contact area of 6 μm^2^; Fig. 4*D*), (*iii*) the measured overall TCR density (*SI Appendix*, Fig. S7), and (*iv*) the fraction of TCRs inside the contacts (40%; Fig. 2*D*). When similar numbers of TCRs engage conventional ligands [∼30 TCRs (10)], signaling is initiated in CD4^+^ T cells, accounting for why TCR triggering is observable for T cells interacting with SLBs (24).

### Self/Nonself Discrimination.

The hallmark of the TCR is its ability to recognize low-density agonist pMHC and to discriminate between weak/self and strong/agonist pMHC. We determined whether, under the simple constraints imposed by our model, the TCR would be capable of discriminatory signaling.

**Fig. 5.**
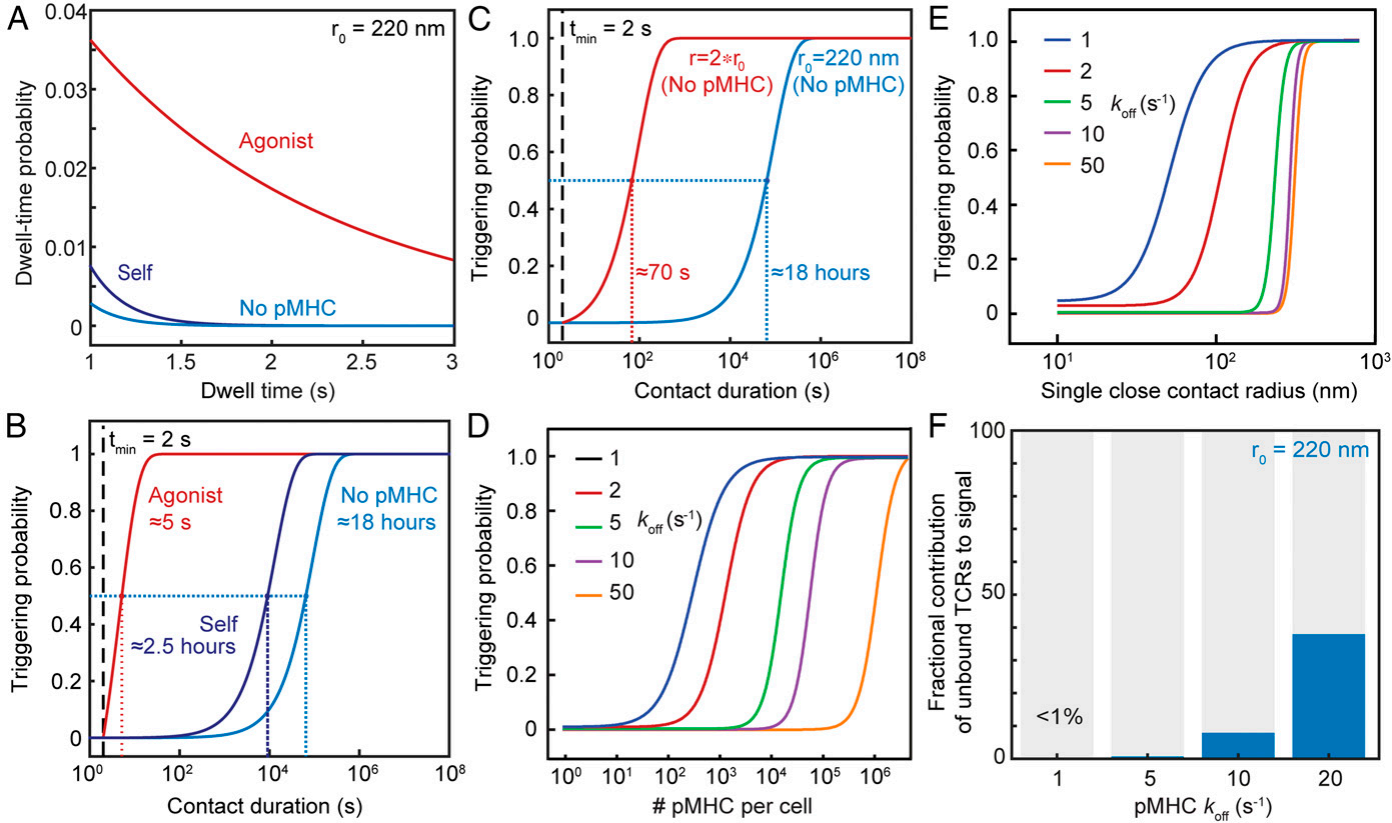
Self/nonself discrimination. (*A*) Probability distribution of close-contact residence times for TCRs in the presence and absence of agonist and self pMHC, for a close contact of radius r_0_ = 220 nm, showing that discrimination of ligands is not dependent on a threshold value for *t*_*min*_. (*B*) Probability that at least one TCR will be triggered, i.e., stay in the contact for *t*_*min*_ ≥ 2 s, as a function of contact duration t_f_ in the presence and absence of agonist pMHC with a low *k*_off_ (*k*_off_ = 1 s^−1^, 30 pMHC/μm^2^), or a self pMHC with a larger *k*_off_ present at higher pMHC densities (*k*_off_ = 50 s^−1^, 300 pMHC/μm^2^); r_0_ = 220 nm. (*C*) Comparison of the triggering probability in the absence of pMHC for close contacts of 220 and 440 nm. (*D*) Triggering probability as a function of pMHC densities and pMHC off rates for a single contact of 220 nm radius with a duration of t_f_ = 120 s. (*E*) Triggering probability as a function of close contact radius for pMHC with varying off rates for a contact duration of t_f_ = 120 s. (*F*) Contribution to the overall signal of TCRs that are triggered without binding to pMHC, in the presence of agonist pMHC with varying *k*_off_ (30 pMHC/μm^2^).

First, we computed the probability distribution of TCR residence times for contacts of r = 220 nm, the size observed when T cells encounter APCs (49, 50). We found that in the absence of ligands, the probability of a TCR remaining inside a close contact for longer than 2 s becomes vanishingly small (Fig. 5*A*): a close contact of this size would need to persist for ∼18 h in order for there to be a 50% probability that a single TCR was triggered (Fig. 5*B*). Strikingly, residence times are much longer for TCRs in the presence of agonist pMHC even at low density (30 pMHC/μm^2^, 2D *K*_d_ given by *k*_on_ = 0.1 μm^2^s^−1^ and *k*_off_ = 1 s^−1^; Fig. 5*A*), which increases the triggering probability ∼12,000-fold, i.e., from 18 h to 5 s (Fig. 5*B*). Residence times were much less affected for pMHC/TCR interactions with self pMHC at relatively high density [300 pMHC/μm^2^ and *k*_off_ = 50 s^−1^, i.e., at the observed low-affinity threshold for nonagonistic TCR/pMHC interactions at high ligand-density (2, 11, 18, 51); Fig. 5*A*], with a 50% TCR-triggering probability requiring contacts of 2.5-h duration (Fig. 5*B*). In other words, a 50-fold increase in *k*_off_, reflecting a very conservative estimate of the lower limit of the *k*_off_ for self pMHC, led to a 1,800-fold reduction in the likelihood of TCR triggering, despite there being 10-fold more self-presenting molecules than agonist pMHCs. This indicates that TCR triggering, based on dwell time at close contacts, would be highly discriminatory. Changes in close-contact size profoundly altered the scope for discriminatory signaling, however. A twofold increase in close-contact radius yielded a ∼1,000-fold increase in the probability of TCR triggering when ligands were absent (*P* = 50% is reached in <70 s versus 18 h; Fig. 5*C*).

Importantly, the model was found to robustly discriminate between ligands of different potency even at low density, a hallmark of TCR triggering. pMHC sensitivity and discrimination were found to be preserved for pMHC densities varying >10^6^ fold, for TCR/pMHC off rates of 1 to 50 s^−1^, and for contact durations t_f_ = 30 and 120 s (Fig. 5*D* and *SI Appendix*, Fig. S8*A*). Discrimination between self and agonist pMHC was optimal for both short- and long-lived contacts between 50 and 300 nm, and lost for contacts larger than 350 nm radius (Fig. 5*E* and *SI Appendix*, Fig. S8*B*). Accordingly, although sensitivity was higher for larger contacts, for smaller *k*_off_ values, and for slower TCR diffusion (*SI Appendix*, Figs. S9 and S10), contacts larger than 350 nm generated significant levels of ligand-independent receptor triggering regardless of ligand levels and TCR behavior, producing the near-complete loss of discrimination (Fig. 5*E* and *SI Appendix*, Fig. S8*B*). The model also predicted that for contacts of 220-nm radius, ligand-independent receptor triggering does not contribute toward overall triggering probability for strong agonist pMHC (*k*_off_ values between 1 and 10 s^−1^; Fig. 5*F*). With increasingly weaker TCR/pMHC interactions (*k*_off_ = 20 and above), the contribution of ligand-independent receptor triggering to the overall triggering probability increased but remained below 50% (Fig. 5*F*). For contacts with ∼220-nm radius, therefore, binding to pMHC is the main determinant of TCR dwell time > *t*_*min*_ inside close contacts.

KP, defined by its dependence on energy-consuming intermediate steps, is often used to explain ligand discrimination by the TCR (13). In some calculations, six intermediate steps are needed to generate >7,500-fold differences in the levels of TCR triggering induced by pMHC ligands differing 10-fold in affinity (13). Such large amplification mechanisms are usually only possible, however, at the expense of sensitivity (13, 15). Our calculations, which simulate a single chemical modification (TCR phosphorylation) and do not rely on a threshold for *t*_*min*_ (Fig. 5*A*), suggest that KP is not required for effective TCR discrimination. A 10-fold difference in affinity produced a ∼1,000-fold difference in TCR triggering for pMHC at densities of 1,000 pMHC/cell (*SI Appendix*, Fig. S8*C*), when close-contact size was restricted (*SI Appendix*, Fig. S8*D*). Even at very low pMHC densities (100 pMHC/cell), there was a ∼100-fold difference in TCR-triggering probability for ligands differing 10-fold in affinity (*SI Appendix*, Fig. S8*C*).

**Fig. 6.**
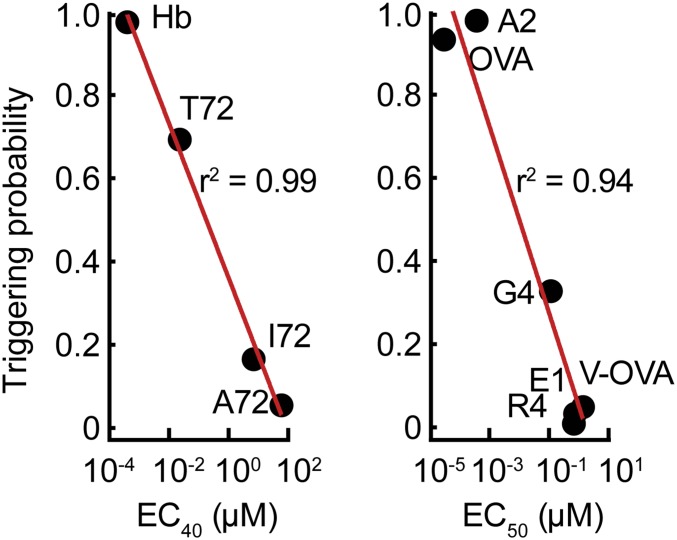
Prediction of the relative signaling potencies of well-characterized TCR ligands. Peptide-stimulation potencies (EC_40_ and EC_50_ values for IL-2 secretion) for CD4^+^ (*Left*) and CD8^+^ T cells (*Right*) (determined elsewhere in refs. 11, 52, and 53), plotted against the probability that at least one TCR triggering event (*t*_*min*_ ≥ 2 s) occurs at a single contact of r_0_ = 220 nm, that persists for t_f_ = 120 s.

Finally, we tested whether the potency of TCR ligands could be correctly predicted, relying only on experimentally determined 2D *k*_on_ and *k*_off_ values (the parameters used are given in Table 1 and *SI Appendix*, Table S2). For 10 different pMHCs and a variety of ligand densities, the calculated TCR triggering probability was found to correlate extraordinarily well with signaling potency measured as IL-2 production in cocultures of peptide-pulsed APCs and T cells (Fig. 6 and *SI Appendix*, Fig. S11; refs. 11 and 52). The correlation was largely unaffected by the use of *k*_off_ values measured under force (10 pN; refs. 11, 53, and 54), which captures catch-bond behavior (*SI Appendix*, Fig. S12*A*). However, at very low ligand densities, and for pMHC with different *k*_on_ but similar *k*_off_ values, catch-bond behavior could rescue the correlation between triggering behavior and IL-2 release, which was otherwise lost (*SI Appendix*, Fig. S12*B*).

## Discussion

We used a quantitative treatment of signaling to explore whether ligand discrimination and sensitivity would be achieved if TCR triggering was governed by receptor dwell time in kinase-containing, phosphatase-depleted close contacts formed when T cells interact with APCs. The model required measurements of (*i*) Lck activity at the levels of CD45/Lck segregation observed at the contacts, (*ii*) TCR density and diffusion, and (*iii*) the size and duration of close contacts. Validating the model in the context of ligand independent triggering, we observed that close-contact growth rate and triggering time were inversely correlated and that signaling was delayed when there was less CD45 segregation and faster when contact area was increased. Our calculations suggested that ligand discrimination and sensitivity would be possible for a triggering mechanism relying only on receptor dwell time at close contacts and that discrimination would not have to be KP-dependent. pMHC-specific responses would then be affected by the kinetics of the TCR/pMHC interaction along with TCR diffusion and T cell topography, since each of these would affect receptor dwell time.

Calculations using the model suggested that signaling outcomes in T cells would be remarkably sensitive to the size of the close contacts they formed. The probability of TCR triggering in the absence of ligands increased dramatically for close contacts with radii beyond the dimensions of contacts observed in vivo (220 nm; refs 47, 49, 50, and 55). For close contacts like those observed in vivo, however, a T cell would need to remain in contact with an APC for almost a day in order for a single TCR to be triggered in the absence of ligands. Thus, even though it is easily demonstrated for larger contacts in vitro (24), it seems unlikely that ligand independent TCR triggering would occur in vivo. Assuming the formation of close contacts with radii at or below 220 nm, we were able to predict the relative potency of pMHC ligands with remarkable accuracy (*r*^2^ = 0.94 to 0.99). The previous best predictions were obtained by Aleksic et al. (56) (*r*^2^ = 0.83), using the concept of “confinement time” (the total time a TCR is occupied by pMHC before complete dissociation). The improved predictive ability of the model likely arises partly due to our use of 2D rather than 3D binding parameters, but mostly because of the spatial constraints imposed by limiting contact size. Our analysis also showed that the level of very early signaling (i.e., ITAM phosphorylation) might be predictive of the scale of a late signaling outcome (IL-2 release). It has been suggested that forces in a cell–cell contact act to reinforce agonist binding (via catch bonds) and destabilize the binding of self pMHC (via slip bonds). We found, however, that the correlation between predicted triggering behavior and IL-2 release was largely unaffected by the use of 2D *k*_off_ values measured under force, except at very low ligand densities.

In contrast to most other receptors, such as G protein-coupled receptors, which are triggered in a largely binary fashion by single ligands, the TCR can react to multiple ligands varying up to 10^6^-fold in affinity (28). The discriminatory ability of the TCR has been proposed to derive from KP (13). In a previous simulation of the KS model, multiple steps producing long delays were required for effective KP because kinase activity was assumed to increase 200-fold inside versus outside close contacts, resulting in even short-lived complexes being phosphorylated (57). Our calculations suggest, however, that discrimination is achievable in the absence of KP, i.e., in a single step—TCR phosphorylation by Lck. This is possible because we assume a relatively modest increase in net kinase activity inside close contacts, based on experimental measurements in this study, which greatly reduces the likelihood that weakly bound receptors will be phosphorylated at small contacts. One interesting possibility that could be explored is that the short residence times of self pMHC/TCR complexes and free TCRs at small contacts also make cells more sensitive to changes in dwell time resulting from agonist pMHC/TCR complex formation. The absence of any requirement for KP explains, at least in part, T cell sensitivity, with our calculations suggesting that ∼200 agonist pMHC/cell would give half-maximal responses.

Our findings have several important implications. First, the size of close contacts committing T cells to synapse formation may have to be tightly controlled to avoid nonspecific activation. Defects in processes that constrain close-contact size could predispose to autoimmunity by increasing ligand-independent receptor signaling. Second, we can explain the extent to which TCR triggering is enhanced by pMHC binding, without the triggering mechanism having to be strictly ligand-dependent. For TCRs interacting with typical ligands at small contacts, we calculated that agonist-dependent signaling is favored as much as 12,000-fold over ligand-independent signaling. Third, some degree of signaling in the absence of ligand might nevertheless explain both TCR polarization and partial TCR phosphorylation (58, 59). We estimated that ∼50% of TCRs remain >0.5 s inside close contacts of ∼220-nm radius, yielding >1 pTyr/contact. This might not initiate downstream signaling but could generate the pMHC-independent, low-level “tonic” TCR triggering observed in vivo (58). Fourth, for close contacts increasing in radius beyond 220 nm, perhaps following an initial round of ligand-dependent signaling, ligand-independent receptor triggering might reinforce or amplify the initial response, enhancing sensitivity. Lastly, the principles established here could be extended to other ITAM-based receptors that are also sensitive to size-based changes in the kinase/CD45 ratio, such as Fc receptors (60–62), or used to calculate the binding “sweet spot” for engineered TCRs (4) or receptor mimics (61).

In conclusion, our work suggests that, rather than KP, topographically constrained T cell contact formation allows, and may even be essential for, ligand discrimination by T cells. The model’s ability to predict the relative signaling potencies of known agonists and nonagonists suggests that it captures the essential features of the TCR triggering mechanism. However, how do T cells ensure that contact size is constrained? So-called T cell “microvilli” are the obvious candidates for achieving this, although further experiments will be required to confirm whether this is true or not. Microvillus-based contacts have radii of 220 ± 20 nm (50) and persist for 1 to 5 min (55, 63, 64). Individual microvillar contacts last >6 s in the absence of cognate antigen, enough time for efficient discriminatory signaling according to our calculations. T cells may thus interrogate their targets using microvilli to exploit their unique topographic properties. Most importantly, our treatment of TCR triggering provides a predictive framework for understanding why it is selective, fast, and sensitive.

## Materials and Methods

A detailed description of the mathematical model of TCR triggering and the experimental procedures for single-particle tracking, superresolution imaging of CD45, quantification of calcium release and close-contact growth, and additional control experiments are provided in *SI Appendix*.

## Supplementary Material

Supplementary File

Supplementary File

Supplementary File

Supplementary File
