# Supplementary material for: A cell topography-based mechanism for ligand discrimination by the T cell receptor
Source: Proc Natl Acad Sci U S A. 2019 Jun 20;116(28):14002–10. doi: 10.1073/pnas.1817255116 (PMC6628812; doi:10.1073/pnas.1817255116)
Supplement: Supplementary File [file pnas.1817255116.sapp.pdf]

Supplementary Information

A cell-topography based mechanism for ligand discrimination by the T-cell receptor

Ricardo A. Fernandes*, Kristina A. Ganzinger*, Justin C. Tzou, Peter Jönsson, Steven F. Lee, Matthieu Palayret, Ana Mafalda Santos, Alexander R. Carr, Aleks Ponjavic, Veronica T. Chang, Charlotte Macleod, B. Christoffer Lagerholm, Alan E. Lindsay, Omer Dushek, Andreas Tilevik§, Simon J. Davis§, David Klenerman§

Contents

I. Appendix I: A quantitative treatment of TCR signaling – description and discussion of the mathematical model

II. Online Materials and Methods

III. Supplementary Tables

Supplementary Table 1 | Quantitative analysis of close contacts formed by T cells interacting with rCD2-presenting SLBs, and organization of key signaling proteins within these regions measured using single-molecule fluorescence microscopy.

Supplementary Table 2 | Parameters used for the mathematical analysis presented in Figs. 3-6.

IV. Supplementary Figures

Supplementary Fig. S1 | Quantification of the CD45 to Lck ratio at different CD45 and Lck densities.

Supplementary Fig. S2 | Quantification of the CD45 to Lck ratio at early close contacts.

Supplementary Fig. S3 | Measurement of TCR diffusion at close contacts formed by T cells interacting with SLBs using single-particle tracking.

Supplementary Fig. S4 | CD45 is evenly distributed on resting T-cell surfaces and segregates from close contacts formed by T cells interacting with glass surfaces, at sub-μm length scales.

Supplementary Fig. S5 | SLB-bound CD45 passively segregates from adhesion protein (rCD2/CD48)-mediated close contacts formed by T cells interacting with SLBs.

Supplementary Fig. S6 | Measurement of HA-CD45 expression levels.

www.pnas.org/cgi/doi/10.1073/pnas.1817255116

Supplementary Fig. S7 | Measurement of TCR numbers on CD48$^+$ Jurkat T-cells.

Supplementary Fig. S8 | The effect of pMHC density and close-contact radius on triggering probabilities for ligands with different k_{off}.

Supplementary Fig. S9 | Combined effect of pMHC density and close contact radius on triggering probability for ligands with different k_{off}.

Supplementary Fig. S10 | Effect of diffusion coefficient on triggering probability for ligands with different k_{off}.

Supplementary Fig. S11 | Effect of pMHC density on predicted T-cell signaling probability based on known 2D TCR/pMHC binding properties.

Supplementary Fig. S12 | Effect of force on predicted T-cell signaling probabilities using known 2D TCR/pMHC binding properties.

V. Supplementary Movies

Supplementary Movie S1 | Animation showing changes in TCR occupation-probability density across a growing close contact over time.

Supplementary Movie S2 | CD45RABC spontaneously segregates from CD2-mediated close contacts formed by CD48$^+$ Jurkat T-cells interacting with CD2- and CD45RABC-presenting SLBs.

Supplementary Movie S3 | Imaging Ca^{2+} release for Jurkat T-cells forming CD45-depleted contacts with labeled-CD2 presenting SLBs.

VI. Additional references

I. Appendix I: A quantitative treatment of TCR signaling

a) Description of the rationale and the assumptions of the TCR triggering model

This model seeks to mathematically describe TCR dwell-time inside close contacts. Close contacts are defined as any area of cell contact from which CD45 is excluded, and therefore correspond to the "close contacts" first described in Chang *et al.* Our experiments and others show that these contacts are initially small and grow over time (see Movie S3; Chang et al., 2016, Razvag et al., 2018). Cell-cell contacts appear to be highly dynamic in the absence of signaling. Since we are interested in signal initiation, we assume that close contacts form for finite periods (referred to as the contact duration).

We base our model on the kinetic-segregation (KS) model (Davis and van der Merwe, 1998; Davis et al. 2006). The KS model proposes that receptor triggering requires only that the TCR stays accessible to kinases within close contacts, protected from phosphatases that would otherwise terminate signaling, and for TCR phosphorylation to be sufficiently long-lived for downstream effects to be initiated. pMHC ligands, *via* trapping effects, serve only to increase the residence time of the TCR inside the close contact. We therefore assume for our modeling (1) that when a close contact is formed, TCRs can diffuse in and out of the contact, and (2) that while the TCR is bound to a ligand inside the close contact, it is unable to leave. The second assumption means that the residence time of a bound TCR is independent from its diffusion, so we do not need to account for any changes in TCR diffusion coefficients upon pMHC binding: the dwell-time of a bound TCR is solely determined by the off-rate of the complex, combined with its free diffusion before and after pMHC binding. The assumption that a pMHC/TCR pair will not leave the contact is supported by the fact that this is energetically unfavorable due to the complex's size being of the same dimensions as the spacing of the two membranes in a close contact (James and Vale, 2012). Irrespective of its binding status, any TCR that remains in the close contact for *2 seconds or longer* – due to pMHC binding per se or due to diffusion, or both – is assumed to be triggered by our model. We chose t_{min} = 2 seconds, given that k_{cat}(Lck) = 3.41 s^{-1} and that, at a CD45/Lck ratio of 2:1, ITAM phosphorylation is reduced by ~50% (Hui and Vale, 2014). We note that the regulatory phosphorylation of Lck (on Y394 and Y505 residues), also under the influence of CD45, has very little effect on k_{cat} and is therefore unlikely to affect this parameter (i.e. unphosphorylated and doubly phosphorylated Lck have similar k_{cat}, 3.41 versus 3.29 s^{-1}). If therefore we assume an effective ITAM phosphorylation rate of $k_{cat,eff} \approx 2\ s^{-1}$, this still means that in 2 seconds ~ 4 pTyr-generating events would occur, which correspond to two pTyr ITAM signaling domains. We further assume that this is sufficient to initiate downstream signaling. There is experimental evidence that TCR triggering occurs within this time-frame upon pMHC binding (2–6). In essence, by introducing t_{min} we consider a distribution of phosphorylation times with a sharp lower boundary created by the maximal turnover rate of Lck in the case of immediate substrate binding (i.e. k_{cat}). If a TCR leaves but re-enters shortly thereafter, then that TCR's sojourn inside the close contact is considered separately with a restarted clock, assuming that triggering occurs after a TCR has occupied the close contact continuously for 2 seconds and not cumulatively over multiple excursions, since CD45 reverses any phosphorylation once the TCR leaves the close contact. The model calculates TCR density across the close contact based on the rate of TCR entry into this area (for a given contact radius, initial TCR density and TCR diffusion coefficient).

Therefore, it can also account for changes in TCR entrance rate and in the dwell-time of TCRs already present inside the close contact as it grows. To achieve sufficient accuracy, the model requires moving-boundary coupled partial differential equations that are computationally expensive. While the problem is solved numerically on a disc, the solution is fully two dimensional and not radially symmetric (although the domain is). Our method of simulation is based on a finite element discretization implemented in MATLAB.

b) Modelling of receptor triggering using moving-boundary coupled partial differential equations to account for close contact growth

See Figs 1-5, Supplementary table 2 and Movie S1

i) Model formulation

Since close contacts (CC) grow on time-scales similar to the diffusion of the TCR, changes in TCR density in CC need to be described by a coupled system of moving-boundary partial differential equations (PDEs),

$$\begin{aligned} \partial T/\partial t &= D_T \nabla^2 T - k^*_{\text{on}} T + k_{\text{off}} C, 0 < |\mathrm{r}| < R(t; t_{\text{entry}}); \\ \partial C/\partial t &= D_C \nabla^2 C + k^*_{\text{on}} T - k_{\text{off}} C, 0 < |\mathrm{r}| < R(t; t_{\text{entry}}), \end{aligned} \qquad \text{(equation 1.1)}$$

where $T(\mathbf{r}, t; t_{\text{entry}})$ and $C(\mathbf{r}, t; t_{\text{entry}})$ represent free and ligand-complexed TCRs diffusing with coefficient $\mathrm{D_T}$ and $\mathrm{D_C}$, respectively, and with the receptors undergoing reversible binding with first-order rates (k^*_{on},$\mathrm{k_{off}}$). Note that $k^*_{on} = k_{\text{on}}[\mathrm{M}]$ where k_{on} is the bimolecular on-rate (in units of $\mu m^2/s$) and $[\mathrm{M}]$ is the ligand concentration (in units of μm^{-2}). We have assumed that TCRs within a CC do not compete for pMHC and this is reflected in using first-order kinetics for binding. This approximation is reasonable when the number of pMHC is larger than the number of bound TCRs at all times within the CC. The boundary conditions for the disc domain of radius R are adsorbing for T and no flux for C,

$$\begin{aligned} T(R) &= 0, \\ D_C \nabla C \cdot \hat{\mathbf{n}} + R'(t) C &= 0 \end{aligned} \qquad \text{(equation 1.2)}$$

Importantly, the domain area grows linearly in time and therefore,

$$R(t; t_{\text{entry}}) = \sqrt{R_0^2 + g(t + t_{\text{entry}})/\pi} \qquad \text{(equation 1.3)}$$

where g is the growth rate (in units of $\mu m^2/s$) and t is time. The initial conditions at $t = t_{entry}$ are as follows,

$$\begin{aligned} T(r) &= \delta(\mathbf{r} - \mathbf{r_0}); \\ C(r) &= 0, \end{aligned} \qquad \text{(equation 1.4)}$$

where $r_0 = (R_0 - \varepsilon, \theta)$. The additional term $R^{'}(t)C$, which reflects the rate of growth in the region, is a necessary addition to the usual Neumann condition in order to prevent mass of C leaving the domain. To see this, consider the change in total mass $M(t) = \int_{\Omega(t)} (T + C)\, d\mathrm{r}$,

$$M'(t) = \frac{d}{dt}\int_0^{2\pi}\int_0^{R(t)} (T + C)\, r dr d\theta \quad = RR'\int_0^{2\pi} [T + C]_{r=R}\, d\theta + \int_0^{2\pi}\int_0^{R(t)} \frac{d}{dt}(T + C)\, r dr d\theta,$$

$$= \int_0^{2\pi} [RR'(T + C) + R(D_T T_r + D_C C_r)]_{r=R}\, d\theta$$

$$= R\int_0^{2\pi} [D_C C_r + R'C]_{r=R}\, d\theta + D_T R\int_0^{2\pi} [T_r]_{r=R}\, d\theta$$

(equation 1.5)

The flux of TCRs in complex (C) through the boundary should be zero which gives rise to the boundary condition. In the case of modeling CC of fixed size (Fig. 4 A-C), $g = 0$ in equation 1.3. The parameter ϵ is the distance from the boundary that the TCR is initialized in its exploration of the CC. For technical reasons, the initial location cannot be exactly on the boundary as one would ideally like since the mathematical formulation of the dwell time would not be well posed. This is because a Brownian walker initially on the boundary will interact with the boundary an infinite number of times. Therefore, we must start the particle just inside the domain and we have used a value of ϵ = 0.09 for the numerical simulations.

ii) Model output

The output of the model is the probability (P_s) that a single receptor has remained within the CC for more than 2 seconds, for contact duration (t_f),

$$P_s(t_{\text{entry}}) = \int_{\Omega(2;t_{\text{entry}})} T(\mathbf{r}, 2; t_{\text{entry}}) + C(\mathbf{r}, 2; t_{\text{entry}})\, d\mathbf{r}. \qquad \text{(equation 1.6)}$$

The time-dependent rate of TCR entry into the domain ($k_t(t)$) is expected to be proportional to the size of the domain, which increases over time. Using previously derived results (see Equation 11 in Weaver, 1983), we find,

$$k_t(t) = \frac{4\pi D T_m}{\log\left(A/(\pi R(t)^2) - 1\right)} \qquad \text{(equation 1.7)}$$

where $A = 415\, \mu m^2$ is the cell surface area, $T_m = 100\, \mu m^{-2}$ (varied over the simulations; see also Table S3) is the TCR density far away from the CC, and $D_T = 0.05\, \mu m^2/s$ (varied over the simulations, see also Table S3) is the TCR diffusion coefficient. With these numbers, we find that the rate of TCR entry into the domain (k_t) increases from $\approx 4\, s^{-1}$ to $\approx 18\, s^{-1}$ as the domain radius increases from 0.01 µm to 2 µm. Note that since we numerically solve the problem by a finite element method, the coordinate singularity at r = 0 does not need to be resolved in this setting.

Given that multiple receptors can enter the CC during the contact duration (t_f), we need to calculate the probability that at least one TCR has remained within the domain for more than 2 s (P_m, referred to as "triggering probability"). The number of TCRs that have entered the domain in time interval $[t_i, t_i + \Delta t]$ can be estimated as $k_t\,(t_i)\Delta t$ so that P_m is estimated as follows,

$$P_m = 1 - \prod_{i=1}^{N}[1 - P_s(t_i)]^{k_t(t_i)\Delta t}. \qquad \text{(equation 1.8)}$$

In the case where P_s and k_t are constants:

$$P_m = 1 - \prod_{i=1}^{N}[1 - P_s]^{k_t\Delta t} = 1 - [1 - P_s]^{k_t N\Delta t} = 1 - [1 - P_s]^{k_t(t_f-2)} \qquad \text{(equation 1.9)}$$

When considering the limiting case of kt(t) = 0 for the growing CC model, we have assumed a small initial radius (0.01 μm) and therefore assumed that the CC is empty of TCRs. In cases where we do not have a growing CC ($g = 0$, Fig. 3C), a term is included representing the initial number of TCRs in the CC at $t = 0$,

$$T_m\pi\, R^2 \times\ P_s(t = 0) + \sum_i k_t(t_i)\Delta t \times\ P_s\,(t_i) \qquad \text{(equation 1.10)}$$

All plots for the theoretical modelling of TCR triggering were generated in MATLAB (MATLAB R 2014b, The MathWorks, Natick, US) using the equations derived in this section.

c) Quantification and statistical analysis

Data were analysed by Graphpad Prism and Origin Lab built-in T-test (unpaired, two tailed), and results were considered significant when p < 0.05. Other statistical parameters including the number of replicates, fold-changes, percentages, SEM, SD, number of cells, number of tracks and statistical significance are reported in the figures, figure legends and supplementary tables.

d) Data and code availability statement

The data sets generated during and analyzed during the current study as well as all custom-written software are available from the corresponding author on reasonable request.

II. Materials and Methods

Cell lines

Human Jurkat T-cells (clone E6-1) and J.RT3-T3.5 cells were obtained from ATCC (ATCC®TIB-152™). All other cell lines were transduced with lentivirus to express the gene of interest. Human HEK293T were obtained from ATCC (ATCC® CRL-3216™). T cells were cultured in sterile RPMI (Sigma Aldrich) supplemented with 10% FCS (PAA), 2 mM L-glutamine (Sigma Aldrich), 1 mM sodium pyruvate (Sigma Aldrich), 10mM HEPES (Sigma Aldrich), and 1% Penicillin-Streptomycin-Neomycin solution (Sigma Aldrich). HEK293T cells were cultured in sterile DMEM (Sigma Aldrich) supplemented with 10% FCS (PAA), 2mM L-glutamine (Sigma Aldrich) and 1% Penicillin-Streptomycin (Sigma Aldrich) at 37°C and 5% CO_2 and were maintained between 10% to 90% confluency. Cells were maintained at 37°C and 5% CO_2 during culturing, and handling was performed in HEPA-filtered microbiological safety cabinets. Typically, cells were kept at a density between 5-9 x 10^5 cells/ml. The UCHT1 hybridoma was a generous gift from Dr Neil Barclay, Sir William Dunn School of Pathology, University of Oxford and the GAP8.3 hybridoma was obtained from ATCC (HB-12™). Hybridomas were cultured in sterile DMEM (Sigma Aldrich) supplemented with 10% FCS (PAA), 2mM L-glutamine (Sigma Aldrich) and 1 mM sodium pyruvate (Sigma Aldrich) at 37°C and 5% CO_2 and were maintained between 10% to 90% confluency. All cells used throughout this study were regularly tested for mycoplasma.

Plasmids

For expressing HA-CD45-Halo, LCK-Halo, and TCRβ-Halo (New England Biolabs, UK) the genes were amplified by PCR to produce dsDNA fragments encoding the proteins of interest flanked at the 3′ end by a sequence coding for a Gly-Ser linker which was followed by Halo-tag. Following confirmation of sequence and reading frame integrity the Lck-Halo and TCRβ-Halo were sub-cloned into the lentiviral pHR-SIN plasmid. To generate mmLck the appropriate residues were mutated by a PCR amplification reaction using forward and reverse oligonucleotides encoding the desired mutation. Sequence integrity was confirmed by reversible terminator base sequencing.

Generation of stable transduced cell lines

Jurkat-derived T-cell lines stably expressing either Lck-Halo or TCRβ-Halo were generated using a lentiviral transduction strategy. HEK293T cells were plated in 6-well plates at 6 x 10^5 cells per well in DMEM (Sigma Aldrich), 10% FCS (PAA) and antibiotics. Cells were incubated at 37℃ and 5% CO_2 for 24h before transfection with 0.5 µg plasmid/well of the lentiviral packaging vectors p8.91 and pMD.G (2nd generation; Addgene) and the relevant pHR-SIN lentiviral expression vector using GeneJuice® (Merck Millipore) as per the manufacturer's instructions. 48 h post transfection, the supernatant was harvested and filtered using a 0.45 µm Millex®-GP syringe filter unit to remove detached HEK293T cells. Three milliliters of the lentiviral-conditioned medium were added to 1.5 x 10^6 Jurkat T-cells.

Fab preparation and labeling

Cell-expressed CD45 was labeled with Alexa Fluor 488 Fab (Gap8.3, anti-CD45; purified from hybridoma supernatant). The Fab was prepared from purified antibody using immobilized papain

(ThermoFisher) per the manufacturer's protocol. Fab digestion and purity were confirmed by size exclusion chromatography. For Fab labeling, Alexa Fluor 488 and Alexa Fluor 647 antibody labeling kits (ThermoFisher) were used as per the manufacturer's protocol. For cell labeling, 1 ml of 5 x 10^5 cells/ml was incubated with Fab (1-10 nM) on ice for 25 minutes. Cells were washed three times in PBS (phosphate buffered saline, pH 7.4).

Fluorescence-activated cell sorting and quantification of protein expression

Wild type or transduced Jurkat and HEK293T cells were washed once in ice-cold PBS, and 1 million cells were incubated with appropriate antibodies (isotype control, eBioscience, UK; Gap 8.3 antibody purified from hybridoma supernatant; anti-HA, clone HA-7, Sigma Aldrich; UCHT1 antibody purified from hybridoma supernatant) at 10 µg/ml for 30 min on ice in PBS/0.05% Azide, washed once in PBS and incubated with a fluorescently labeled secondary anti-mouse antibody as appropriate [Alexa Fluor-647 or -488 conjugated (Molecular Probes, Invitrogen), or PE-conjugated antibody (Sigma Aldrich)] for a further 30 min on ice. Cells were washed in ice-cold PBS and analysed on a Beckman Coulter CyAn Analyser. For quantification of cell surface protein expression, QuantiBrite-PE Beads (BD Biosciences) were used as per the manufacturer's instructions. Mean fluoresence intensity analysis and further data processing was performed with FlowJo software using a standard gate on live cells based on the forward- and side-scatter profile.

HaloTag® labeling

Cells expressing HaloTag® (Promega, UK) fusion protein (Lck, TCR) were labeled with TMR Cell* following the manufacturer's preparation protocol (www.promega.co.uk/products/imaging-and-immunological-detection/cellular-imagingwithhalotag/proteintrafficking). First, the cell medium was replaced with 200 µl RPMI without supplements to which 1-5 µM of Halo-Tag TMR dye was added, and the cells then incubated at 37°C for 45 minutes. To ensure that free dye would not remain in the cytoplasm, cells were washed three times in HBS and then further incubated at 37°C for 30 minutes followed by another three washes with PBS.

Sample preparation for calcium response measurements

Jurkat T-cells were labeled with 4 µM Fluo-4 AM (F-14201; Invitrogen, UK) for 30 min at room temperature with 2.5 mM probenecid (P-36400; Invitrogen, UK) in RPMI (Sigma-Aldrich, UK) without supplements. Cells were then washed in HBS (51558; Sigma, UK) and the medium changed to HBS containing 2.5 mM probenecid before their addition to the microscope sample container with the prepared microscope coverslip.

Total internal reflection microscopy (TIRFM)

For all Figures containing TIRFM data (except Fig. S4 B,C): TIRFM imaging was performed using total internal reflection fluorescence microscopy (TIRFM). A diode laser operating at 488 nm (20 mW, Spectra Physics, US) and either a diode laser operating at 561 nm (Excelsior, 20 mW, Spectra Physics, US) or a HeNe laser operating at 633 nm (25LHP991230, Melles Griot) were directed into a TIRFM objective (60x Plan Apo TIRF, NA 1.45, Nikon Corporation, Japan) mounted on an Eclipse TE2000-U microscope (Nikon Corporation, Japan) parallel to the optical axis and offset in order to

achieve total internal reflection of the beam. The emitted fluorescence was collected by the same objective and separated from the returning TIR beam by a dichroic mirror (FF500/646-Di1, 488/633 emission, Semrock, US) or (XF2044-490-575DBDR, 488/561 emission, Omega Optics). The green fluorescence emission was subsequently separated from the red fluorescence emission by a second dichroic mirror and filter sets; for excitation at 488 and 633 nm: FF605-Di02 (Dual-View mounted, Photometrics, Roper Scientifics, US), FF03-525/50-25 (488 emission), BLP01-635R-25 (633 emission), all Semrock, US; for excitation with 488 and 561: FF562-Di03 (Dual-View mounted, Photometrics, Roper Scientifics), FF02-525/40-25 (488 emission), LP02-568RS-25 (561 emission), all Semrock, US. The fluorescence signals from both channels were simultaneously recorded using an EM-CCD camera (Cascade II: 512, Photometrics, Roper Scientifics, US) operating at -70 °C, whereby each color was recorded on one half of the EMCCD chip. A grid consisting of regularly spaced ion-beam etched holes in gold-on-glass was used to achieve image registration across both emission channels. The Dual-View optics were adjusted to maximize the overlap of the grid images in the two channels under bright field illumination, resulting in a mean alignment precision of approximately 120 nm. Data were acquired using either single snap shots or time-lapse acquisition using Micromanager (Edelstein et al., 2010).

For Fig. S4 B,C: A collimated 640 nm (LaserBoxx 641, Oxxius, Lannion, France) laser beam was focused at the back aperture of a 60x oil TIRF objective (Olympus, NA 1.49, UIS2 series APON 60XOTIRF) mounted on an IX71 Olympus inverted microscope frame. The power of the collimated beams at the back aperture of the microscope was 20 mW. Emitted fluorescence was collected by the same objective, separated from the excitation by a dichroic (Di01-R4-5/488/561/635, Semrock), expanded through a 2.5x achromatic beam expander (Olympus, PE 2.5x 125) and sent through an emission filter 635 long-pass filter (Semrock, BLP01-635R). The images were recorded on an EM-CCD camera (Evolve 512, Photometrics) operating in frame transfer mode at -80°C. Videos of 1000 frames were acquired at exposure times of 30 ms using Micromanager.

Double-helix Point-spread function (DHPSF) microscopy

As previously described (Carr et al., 2017), DHPSF imaging was carried out on a bespoke microscope incorporating a 1.27 NA water immersion objective lens (CFI Plan Apo IR SR 60XWI, Nikon) mounted onto a scanning piezo stage (P-726 PIFOC, PI) on a conventional fluorescence microscope body (Eclipse Ti-U, Nikon). A 4*f* system of lenses with a 650 nm optimized double-helix phase mask (DoubleHelix, Boulder USA) placed in the Fourier-plane performed the DHPSF transformation and relayed the image onto an EMCCD detector (Evolve Delta 512, Photometrics). Collimated 640 nm (200 mW, iBeam smart-640-s, Toptica) and 405 nm (120 mW, iBeam smart-405-s, Toptica) lasers were directed into the objective lens, resulting in a power density at the sample of ~1 kW/cm^2 and 100 W/cm^2 respectively. The fluorescence signal was separated from the laser excitation by a quad-band dichroic filter (Di01-R405/488/561/635-25x36, Semrock). Additional isolation of the fluorescence signal was achieved by long-pass and band-pass filters placed directly before the detector.

Supported lipid-bilayer (SLB) preparation

Prior to SLB formation on glass cover slips, the cover slips (size no. 1: 0.13 mm in thickness, VWR International, UK) were cleaned by incubation in Piranha solution (3:1 sulfuric acid:hydrogen peroxide) for 1 hour followed by thorough rinsing with ultrapure water (MilliQ, 18.2 MΩ resistance) and subsequent plasma cleaning for 15 minutes (Argon, PDC-002, Harrick Plasma). Lipid vesicles were prepared by extrusion through a 50 nm membrane (Whatman, Maidstone, UK) using an Avanti Mini-Extruder (Avanti Polar Lipids, Alabaster, AL). The vesicles consisted of 1-palmitoyl-2-oleoyl-sn-glycero-3-phosphocholine (POPC) with either 0 or 5.0 wt% of 1,2-di-(9Z-octadecenoyl)-sn-glycero-3-[(N-(5-amino-1-carboxypentyl)imino-diaceticacid)succinyl] (nickel salt) (18:1 DGS-NTA(Ni), both Avanti Polar Lipids, Alabaster, USA). POPC vesicles containing 0.01 wt% Oregon Green 488 1,2-dihexadeca-noylsn-glycero-3-phosphoethanolamine (OG-DHPE) were also used for some of the experiments in this work. The buffer solution used in the experiments was HBS buffer (10 mM 2-[4-(2-hydroxyethyl)piperazin-1-yl]ethanesulfonic acid (HEPES), 150 mM NaCl), adjusted to a pH of 7.4, filtered through a 0.2 µm membrane (AnaChem, Lutton, UK) before use. Press-to-seal silicone isolators (4.5 mm diameter, Grace Bio-Labs, Bend, Oregon, USA) were pressed on the cleaned cover slips, following the manufacturer's instructions, to form SLBs within the silicon wells of the isolator by adsorption and subsequent rupture of the lipid vesicles prepared by placing a 20 µl drop of the vesicle solution (1 mg/mL lipids) on the glass surface. After the formation of an SLB from the lipid vesicle suspension (~30 min) the vesicle solution was first replaced with buffer solution and then with a solution containing 0.25 µg/mL of rCD2-6His-spacer-6His. For some experiments, the rCD2 molecules were labeled with Alexa Fluor 488 or 647 (Molecular Probes, Invitrogen, UK) *via* surface exposed lysines. Ni^{2+}-NTA lipids were incubated with rCD2 in the SLB for 60 minutes after which the binding of proteins had reached equilibrium.

Sample preparation for imaging (TIRFM)

Before imaging, approximately 10^6 cells were resuspended in PBS and incubated in a microcentrifuge tube with the desired antibody fragments for 30 min at room temperature (22°C). After the incubation step, the cells were washed three times with PBS by centrifugation and resuspension of the pellet (600×g, 2 min). After the slides were transferred to the microscope stage, the cells were added; imaging was carried out within the first minutes following cell attachment at room temperature.

Sample preparation for imaging (DHPSF)

Jurkat $CD48^+$ T-cells were labelled with Alexa 647 anti-CD45 antibody (Gap8.3) as previously described before being fixed in 4% paraformaldehyde (Sigma-Aldrich) and 0.2% glutaraldehyde (Sigma-Aldrich) for 60 minutes at room temperature. The fixed cells were washed three times in PBS and suspended in GLOX STORM buffer (PBS supplemented with 50 mg/ml glucose [Sigma-Aldrich), 0.02-0.05 mg/ml catalase (Sigma-Aldrich), 0.8 mg/ml glucose oxidase (Sigma-Aldrich) and 7 mg/ml MEA (Sigma-Aldrich)]. Coverslips (24 × 50 mm borosilicate, thickness no. 1, VWR International, UK) were coated with poly-L-lysine (Sigma-Aldrich, Mol wt 150-300 kDa) for 10 minutes, before a 1:100 dilution of 100 nm gold nanoparticles (Sigma-Aldrich) was added for 2 minutes. Coverslips were washed three times with PBS and 50 µL of the fixed cells were placed onto

the coated coverslips. Fixed T-cells were imaged for 200,000 frames with continuous 640 nm and 405 nm HiLo laser excitation and a 30 ms exposure (Tokunaga, et al., 2008). After reconstruction, a rolling-mean of the fiducial marker's position over 50 frames was used to correct for drift in x, y and z.

Image analysis (TIRFM experiments)

Image analysis was performed using a combination of manual analysis (ImageJ, U. S. National Institutes of Health, Bethesda, Maryland, USA, http://imagej.nih.gov/ij/) and custom-written MATLAB code (MATLAB R 2014b, The MathWorks, US).

i) Image acquisition and analysis for single-molecule tracking experiments at close contacts on SLBs

Videos were obtained at a frame rate of 28.6 frames per second (exposure time: 33ms) simultaneously visualizing the distribution of wild-type CD45 (labeled with high Fab concentrations as described above) in one channel and trajectories of either wtCD45, HA-CD45-Halo, mmLck-Halo or TCRβ-Halo in the second channel. Videos were analyzed using custom-written software (MATLAB). An interactive user interface allowed the user to select boundaries for each cell based on bright field images acquired during the data collection phase. Binary masks of the CD45 distribution for the selected cells were obtained by an average Z-projection over 200 frames in the CD45 channel and applying an intensity threshold to the image (pixels were assigned a value of 1 if their intensity values, I, fulfilled the condition $I > (\mathrm{mean}(I_{region}) - 2 * \mathrm{std}(I_{region}))$. The videos for the second channel (recording the trajectories of single molecules in the close contacts) were analysed using the custom-written software (MATLAB) described in Weimann et al. 2013. The positions of the trajectories obtained were then mapped onto binary representations of the contacts created previously using a custom written MATLAB routine. Using these binary images, the MATLAB routine also sorted the trajectories into two categories ("confined within" and "outside" the contact). The diffusion coefficients for these two categories of trajectories were extracted as described elsewhere (Weimann et al. 2013). The number of classified trajectories for a given cell was normalized against the corresponding mask area, i.e. the area "inside" and "outside" the contact, respectively.

ii) Image acquisition and analysis for simultaneous imaging of CD45 distribution in the close-contacts on SLBs and Ca^{2+} signaling (Fluo-4 fluorescence increases) with TIRFM

Data (videos) were acquired on the TIRF microscope as described above at 37°C, using the 488 and 633 lasers for excitation and the corresponding dicroics and emission filters. Data were acquired using alternating excitation and time-lapse acquisition, with an exposure time of 100 ms, and a time between frames of 2 s. The videos were analyzed using custom-written MATLAB code. Briefly, cell positions were manually traced in the last frame of the Fluo-4 channel, and for each cell its mean intensity trace across the entire image sequence was calculated. The time of landing (t_{land}) and Ca^{2+} release (t_{Ca}) were determined in a semi-automated fashion from these intensity traces; t_{land} was determined manually from the bright field image and t_{Ca} automatically by finding peaks in the derivative of the intensity trace. The time taken to trigger was defined as t_{Ca}-t_{land}. For the interval t_{land} to t_{Ca}, the area of cell-surface contact was calculated from the CD45 channel for each cell by

manually tracing the area inside the close contact outlined by CD45 fluorescence in a program-integrated GUI. From this analysis, we obtained the change of cell-surface contact area for individual cells until the time point of Ca^{2+} release, t_{Ca}. All further statistical analysis and fits of the traces were performed with Origin (OriginPro 9.1 G, OriginLab Corporation, Northampton, USA).

iii) Super-resolution microscopy of CD45 distribution in Jurkat T-cells

CD45 was labeled with Gap8.3 conjugated to the fluorescent dye Atto655 and made to emit intermittently by the addition of ascorbic acid (100 µM; for details see Vogelsang et al., 2009). Videos of isolated fluorescent puncta could thus be obtained whose center positions were extracted using the software PeakFit (www.sussex.ac.uk/gdsc/intranet/microscopy/imagej/smlm_plugins), an ImageJ plug-in for super-resolution analysis. Briefly, local maxima in each frame were fitted with a 2D-Gaussian described by seven parameters (position on two axes, standard deviation on two perpendicular axes and angle to the horizontal axis, amplitude, and offset). Finally, each single-molecule position was re-plotted using a custom macro written in ImageJ (http://rsb.info.nih.gov/ij/) as a 2D Gaussian profile defined by the measured integrated intensity and a width given by the average statistical error in localization of the center (95% confidence interval, averaged over all single-molecule localizations); for further details see Ptacin et al., 2010.

iv) DHPSF experiments

All DHPSF data was analysed by easy-DHPSF software (Lew et al., 2015) and rendered using ViSP (Beheiry et al., 2013).

III. Supplementary Tables

Table S1 Quantitative analysis of close contacts formed by T cells interacting with rCD2-presenting SLBs, and organization of key signaling proteins within these regions measured using single-molecule fluorescence microscopy.

Values were obtained from the types of experiments shown in Fig. 2. The automated procedure used to analyze the images and characterize the cell/SLB contacts is described in the Online Methods.

	# Cells/ contacts	Average contact area (μm^2)	Average contact radius (µm)	Confined[1]/ total molecules	Confined molecule density / total density[2]	Diffusion coefficient (mean±s.d. of distribution measured; $\mu m^2/s$) Inside of contact	Outside of contact
**CD45**	39	25±20	2.8±2.6	65/969	0.13±0.03	N.D.[3]	0.113 ±0.179
**Lck**	16	16±21	2.2±2.6	234/1527	0.56±0.07	0.151 ±0.294	0.104 ±0.185
**TCR**	16	13±12	2.1±2.0	48/375	0.40±0.06	0.054 ±0.107	0.050 ±0.074

[1] Trajectories were classified as "confined" if they could be mapped exclusively onto the inner area of a contact (denoted by "1" in the binary representation of the contacts, c.f. Fig. 2B-D, *right panels*).

[2] Ratio of a molecule's density inside the contact over its overall density in the visible region of the cell.

[3] N.D., not determined.

Table S2 Parameters used for the mathematical analysis presented in Figs. 3-6.

<table>
<tr><th></th><th>Fig. 4A</th><th>Fig. 4B</th><th>Fig. 4C</th><th>Fig. 3A</th><th>Fig. 3B</th><th>Fig. 5A</th><th>Fig. 5C</th><th>Fig. 5B</th><th>Fig. 5F</th><th>Fig. 5D</th><th>Fig. 5E</th><th>Fig. 6</th></tr>
<tr><td>Cell surface area (μm^2)</td><td colspan="12">415[5]</td></tr>
<tr><td>Number of close contacts</td><td colspan="12">1</td></tr>
<tr><td>Close contact radius (nm)</td><td colspan="5">Variable[1]</td><td>220[3]</td><td>220[3]/ 440[3]</td><td>220[3]</td><td>220[3]</td><td>220[3]</td><td>Variable</td><td>Variable</td></tr>
<tr><td># TCR copies/cell</td><td colspan="12">41,500[2]</td></tr>
<tr><td># CD45 copies/cell</td><td colspan="12">200,000[2]</td></tr>
<tr><td># Lck copies/cell</td><td colspan="12">40,000[2]</td></tr>
<tr><td>Fraction of TCR segregation</td><td colspan="12">0.63[2]</td></tr>
<tr><td>CD45:Lck ratio</td><td colspan="4">2.5:1</td><td>Variable</td><td colspan="7">2.5:1</td></tr>
<tr><td>TCR diffusion ($\mu m^2/s$)</td><td colspan="12">0.052</td></tr>
<tr><td>k_{on} (pMHC)</td><td colspan="5">N.A.[7]</td><td>0.1</td><td>N.A.</td><td colspan="2">0.1</td><td>0.1</td><td>0.1</td><td>variable</td></tr>
<tr><td>Agonist pMHC (molecules/μm^2) / k_{off}</td><td colspan="5">N.A.</td><td>30 / 1</td><td>N.A.</td><td>30 / 1</td><td>30 / 1</td><td>Variable /1</td><td>30/1</td><td>30 / variable</td></tr>
<tr><td>Non-agonist pMHC (molecules/μm^2) / k_{off}</td><td colspan="5">N.A.</td><td>30 / 50</td><td>N.A.</td><td>300 / 50</td><td>300 /variable</td><td>Variable</td><td>30/variable</td><td>30 / variable</td></tr>
<tr><td>Residence time (s)</td><td colspan="5">N.A.</td><td>result</td><td colspan="6">N.A.</td></tr>
<tr><td>t_{min} (s)</td><td>N.A.</td><td colspan="3">2[3]</td><td>2[3]/20</td><td>N.A.</td><td colspan="6">2[3]</td></tr>
<tr><td>Fraction of triggered TCRs</td><td>N.A.</td><td>Result</td><td>N.A.</td><td>Result</td><td>0.5[4]</td><td>N.A.</td><td colspan="2">N.A.</td><td>Result</td><td colspan="3">N.A.</td></tr>
<tr><td>T cell/APC contact duration</td><td colspan="5">120 s</td><td>N.A.</td><td colspan="2">Variable</td><td>120 s</td><td>120 s</td><td>120 s</td><td>120 s</td></tr>
</table>

[1] Growth rate, g ($\mu m/s$).
[2] Experimentally determined for $CD4^+$ T-cells isolated from PBMCs.
[3] Literature values (see main text).
[4] Arbitrary value used to plot linear relationship.
[5] (Weaver, 1983).
[7] N.A., not applicable.

IV. Supplementary Figures

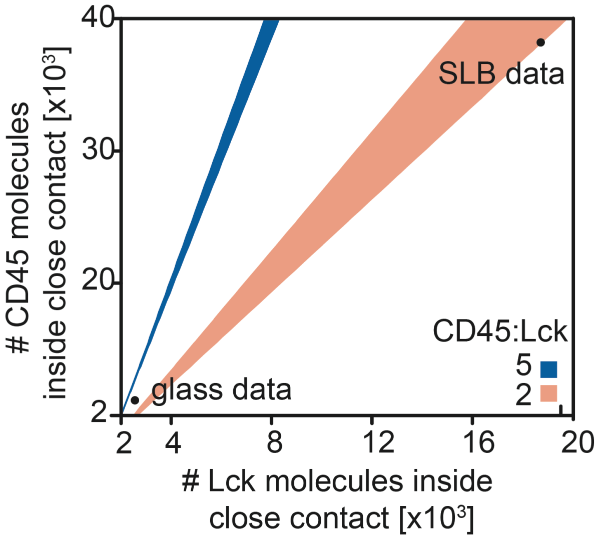

Fig. S1 Quantification of the CD45 to Lck ratio at different CD45 and Lck densities. Graphical depiction of the CD45 to Lck ratios of 5 (blue) and 2 (orange) for varying numbers of CD45 and Lck molecules in the T-cell membrane (±0.25). The single-molecule quantification of CD45 and Lck inside the close contacts (see Figs. 2, S2; and ref. 24 in the main text) indicates that the ratio of CD45 to Lck in the close contact is 2.3-2.7.

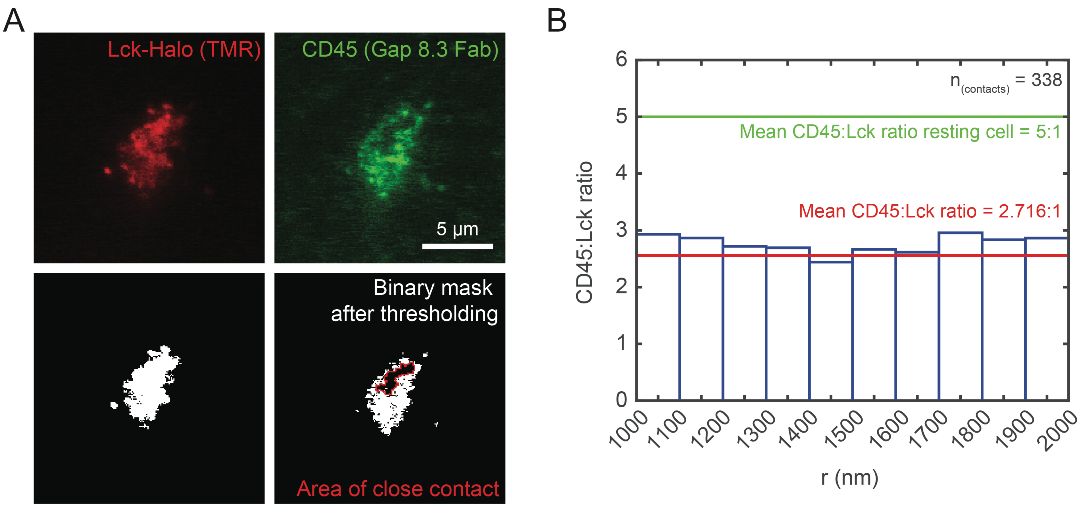

Fig. S2 Quantification of the CD45 to Lck ratio at early close contacts. (*A*) representative frame of a TIRFM video showing the distribution of CD45 (labelled with Gap 8.3 Fab, Alexa Fluor 568) and Lck (labelled with a Halo-tag,TMR) across regions of close contacts of $CD48^{+}$ Jurkat T-cells on rCD2-presenting SLBs. A MATLAB script was used to binarize the image and calculate the ratio of intensities inside (central region of low CD45 intensity) and outside (region of high CD45 intensity) the close contacts (24). (*B*) Assuming copy numbers of 200,000 for CD45 and 40,000 for Lck, the histogram shows the CD45:Lck ratio calculated from the relative intensities as a function of close contact size (i.e. size of the central region of low CD45 intensity). Data is for 338 individual contacts, taken from contact formation videos for 10 cells in 6 independent experiments.

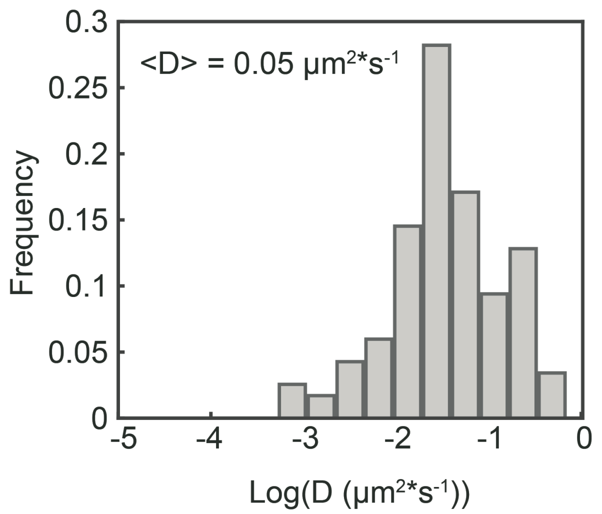

Fig. S3 Measurement of TCR diffusion at close contacts formed by T cells interacting with SLBs using single-particle tracking. Histogram of diffusion coefficients, D, obtained from linear fits (5 points, interval 35 ms) of mean-square displacement of single TCRs (data derived from the type of experiment shown in Fig. 2D). Trajectories for CD45 and Lck were analysed analogously to calculate their diffusion coefficients; the diffusion coefficient cited in Supplementary table 1 is the mean (<D>) and the error the s.d. of the distribution of D.

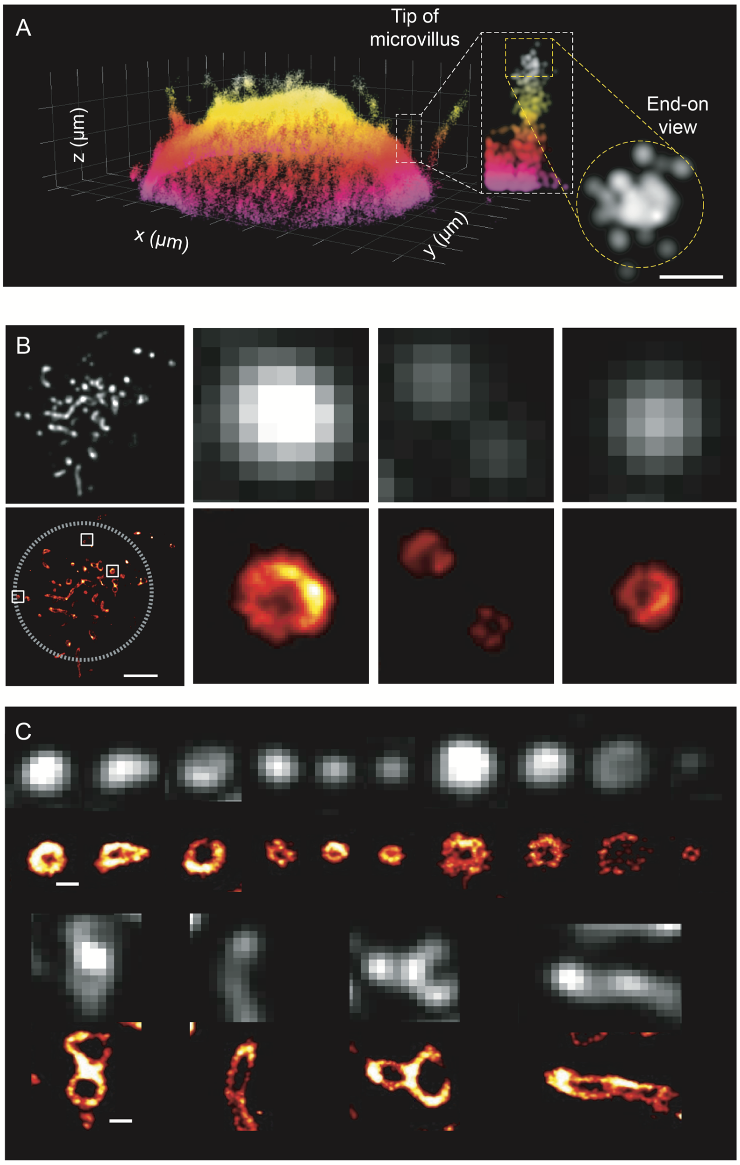

Fig. S4 CD45 is evenly distributed on resting T-cell surfaces and segregates from close contacts formed by T cells interacting with glass surfaces, at sub-μm length scales. (*A*) 3D super-resolution imaging (7) of fluorescently labelled CD45 in the Jurkat T-cell membrane (*left*) and side and tip of a microvillus (250 nm thick section, end-on view, *right*); localization precision is ~15 nm laterally and ~30 nm axially. (*B,C*) 2D super-resolution *d*STORM image of CD45 in early close contacts formed with IgG-coated glass surface; average (*top*) and reconstructed image (*bottom*). (*B*) Representative image of a single T-cell (scale bar 3 μm) with three marked contacts shown at greater magnification on the right hand side. (*C*) Gallery of 20 individual close-up views of close contacts sampled across > 5 cells (scale bar 100 nm).

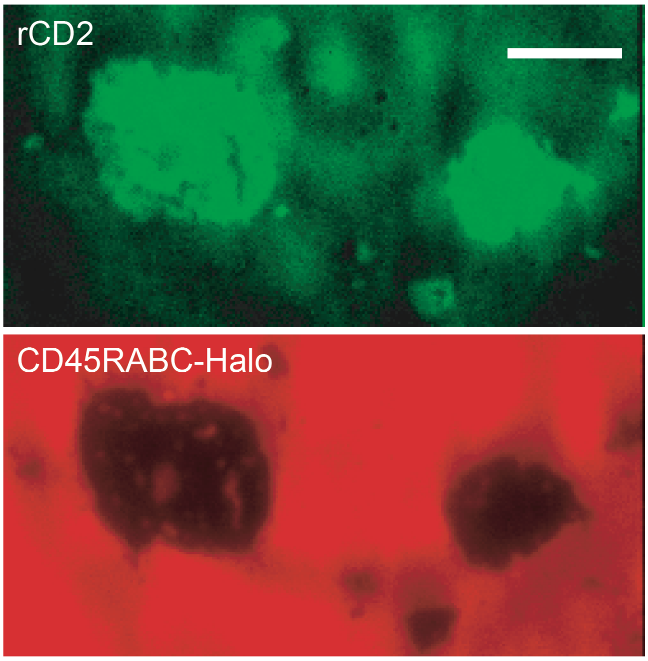

Fig. S5 SLB-bound CD45 passively segregates from adhesion protein (rCD2/CD48)-mediated close contacts formed by T cells interacting with SLBs. The spatial organization of His-tagged rCD2 (Alexa Fluor 488-labeled, green, 1000 molecules/μm^2, top) and His-tagged CD45RABC-Halo (TMR labeled, red, bottom) in contacts of CD48$^+$ Jurkat T-cells and Ni^{2+}-NTA presenting SLBs containing His-tagged rCD2 and His-tagged CD45RABC-Halo (4:1), eight minutes after cell contact with the SLB.

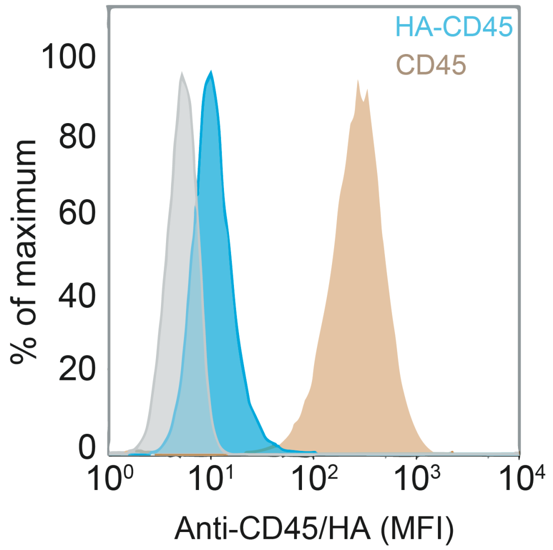

Fig. S6 Measurement of HA-CD45 expression levels. Mean fluorescence intensity of fluorescent anti-HA (clone HA-7, Sigma) or anti-CD45 (Gap 8.3) antibody-stained Jurkat T-cells transduced to express HA-CD45. Anti-HA antibody staining of untransduced Jurkat T-cells is shown in gray.

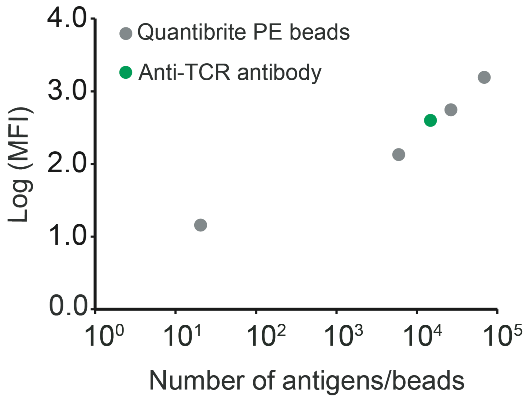

Fig. S7 Measurement of TCR numbers on CD48^{+} Jurkat T-cells. Quantification of the number of TCRs per cell, based on measuring the mean fluorescence intensity of bound PE-labeled anti-TCR antibody and Quantibrite PE beads by FACS.

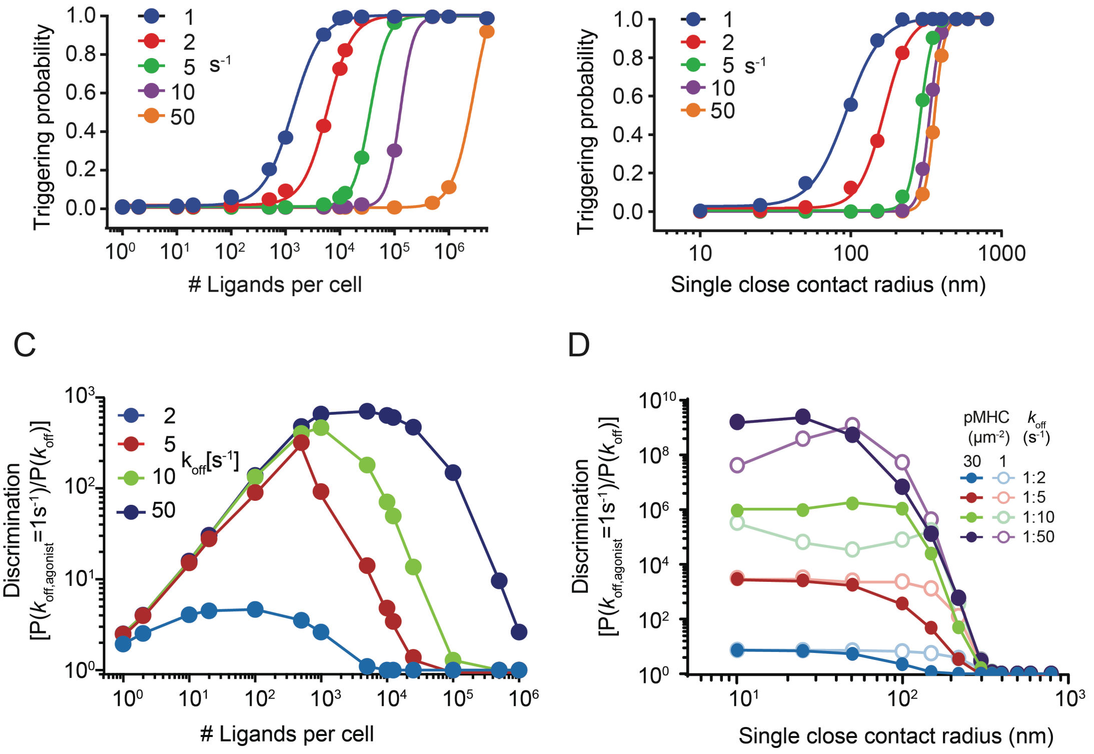

Fig. S8 The effect of pMHC density and close-contact radius on triggering probabilities for ligands with different k_{off}. (*A*) Triggering probability as a function of pMHC density, i.e. ligands per cell, and pMHC off-rate (k_{off}, s^{-1}) for a single contact of 220 nm radius and duration t_f = 30 s. (*B*) Triggering probability as a function of close contact radius and pMHC off-rate for a contact of duration t_f = 30 s. (*C*) Discrimination, i.e. relative triggering probability versus an agonist of k_{off} = 1 s^{-1}, for pMHC ligands (k_{off} = 2, 5, 10 and 50 s^{-1}) forming TCR/pMHC complexes at different pMHC densities. (*D*) Discrimination as a function of close contact radius, r_0, for various pMHC ligands (k_{off} = 2, 5, 10 and 50 s^{-1}) and various pMHC densities.

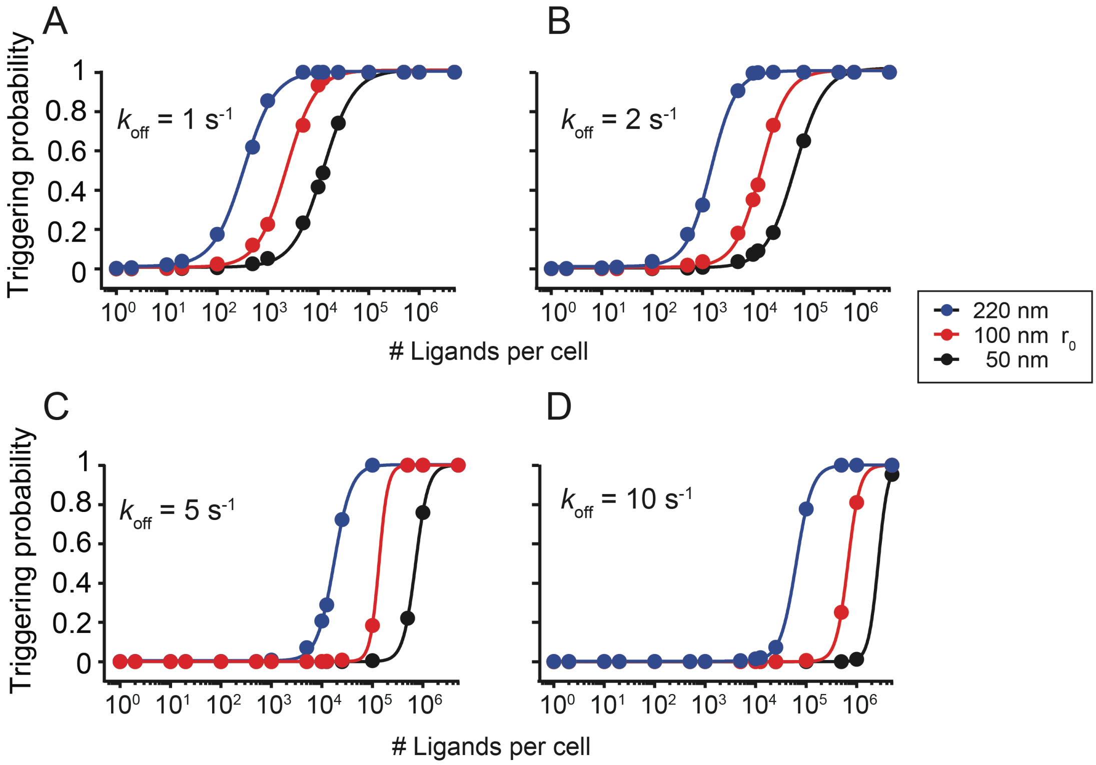

Fig. S9 Combined effect of pMHC density and close contact radius on triggering probability for ligands with different k_{off}. Triggering probability as a function of the number of pMHC ligands per cell for three contact radii r_0 = 50, 100 or 220 nm, and for k_{off} of 1 s^{-1} (*A*), 2 s^{-1} (*B*), 5 s^{-1} (*C*), 10 s^{-1} (*D*), and contact duration t_f = 120s.

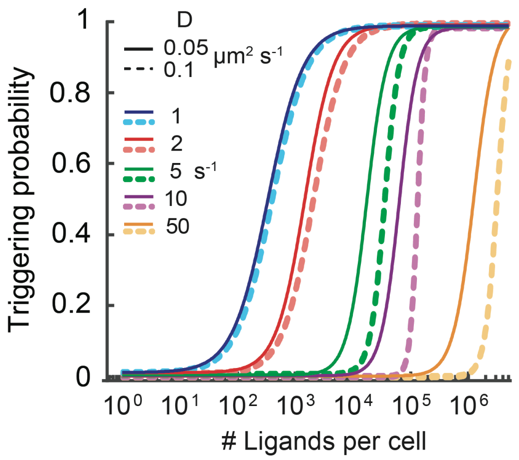

Fig. S10 Effect of diffusion coefficient on triggering probability for ligands with different k_{off}. Triggering probability as a function of ligand density and pMHC k_{off}, for TCRs with diffusion coefficients D = 0.05 μm^{2}s^{-1} and D = 0.1 μm^{2}s^{-1}. The overall trend is preserved with triggering probabilities being shifted to slightly larger peptide densities, in particular for peptides with larger k_{off} values ('self' peptides).

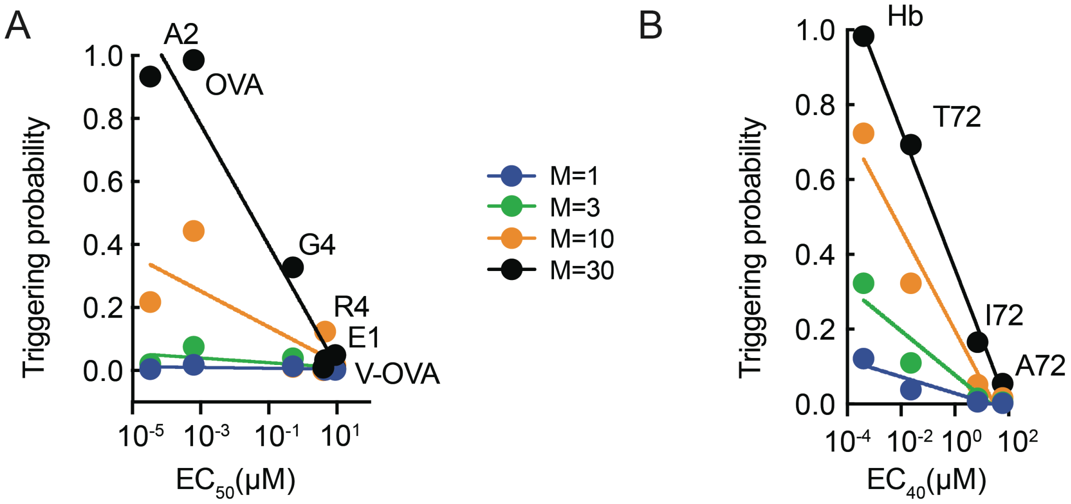

Fig. S11 Effect of pMHC density on predicted T-cell signaling probability based on known 2D TCR/pMHC binding properties. Peptide stimulation potencies, i.e. EC_{40} and EC_{50} values for IL-2 secretion, for $CD8^+$ (*A*) and $CD4^+$ T-cells (*B)* determined elsewhere (refs 8–10), are plotted against the probability that at least one TCR triggering event ($t_{min} \geq 2$ s) occurs at a single contact of r_0 = 220 nm radius that persists for t_f = 120 s, for various pMHC densities (M, ligands/μm^2).

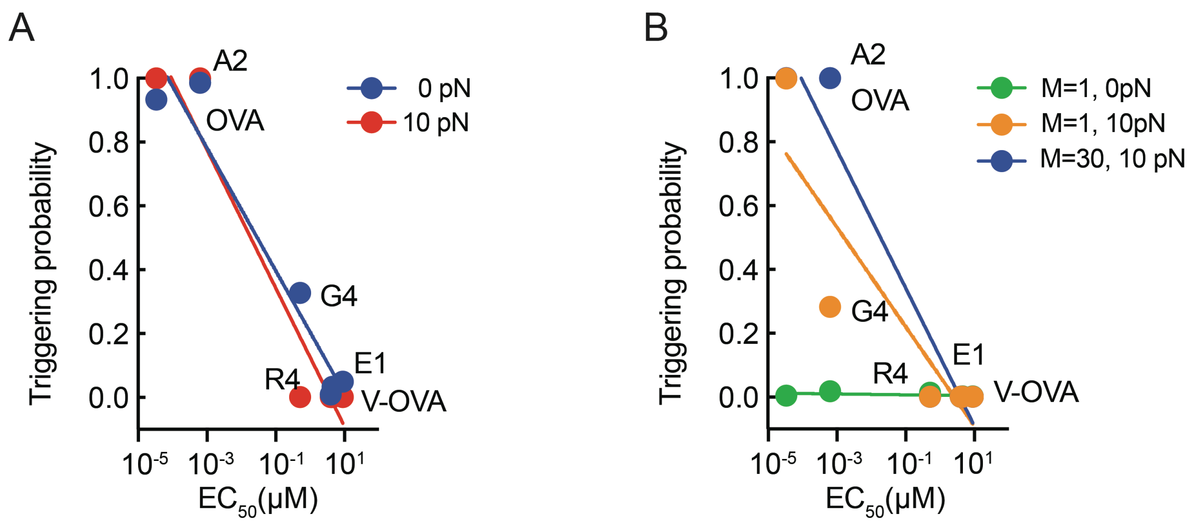

Fig. S12 Effect of force on predicted T-cell signaling probabilities using known 2D TCR/pMHC binding properties. Peptide stimulation potencies (EC_{40} and EC_{50} values for IL-2 secretion), for $CD8^+$ T-cells (determined elsewhere, see refs 8, 11), plotted against the probability that at least one TCR triggering event ($t_{min} \geq 2$ s), occurs at a single contact of r_0 = 220 nm radius that persists for t_f = 120 s, in the presence or absence of force (pN) (*A*) and at 10 pN force for two pMHC densities (M, molecules/μm^2) (*B*).

V. Supplementary Movies

Movie S1

Animation showing changes in TCR occupation-probability density across a growing close contact over time. The probability of occupation is plotted on the z-axis, the 'time' given in the title is the time post initial contact, and the 'remaining mass' refers to the probability that the TCR is still found within the close contact. Close-contact growth rate is set to g = 0.1 $\mu m^2/s$.

Movie S2

CD45RABC spontaneously segregates from CD2-mediated close contacts formed by $CD48^+$ Jurkat T-cells interacting with CD2- and CD45RABC-presenting SLBs. Representative video showing simultaneous rCD2 accumulation and CD45RABC-Halo segregation from stable cell/SLB contacts for $CD48^+$ Jurkat T-cells interacting with a rCD2- and CD45RABC-Halo presenting SLB (rCD2:CD45RABC-Halo ratio of 4:1). The video combines raw data for the CD2 channel (i.e. Alexa Fluor 488-tagged CD2 fluorescence, green; left) with a simultaneously-acquired video of the CD45RABC-Halo channel (TMR labelled CD45RABC, red; right). The video plays 10-fold faster than real-time (5 frames per second). Scale bar, 5 μm.

Movie S3

Imaging Ca^{2+} release for Jurkat T-cells forming CD45-depleted contacts with labeled-CD2 presenting SLBs. Video collage for a $CD48^+$ Jurkat T-cell; the video combines raw data for the CD45 channel (i.e. CD45 labeled with Alexa Fluor 568-tagged Gap 8.3 Fab (red; left) with a video of the Fluo-4 channel (green; right). The video plays 10-fold faster than real-time (5 frames per second). Scale bar, 5 μm.

VI. Additional references

M. El Beheiry, M. Dahan, ViSP: representing single-particle localizations in three dimensions. *Nat. Methods* **10**, 689–690 (2013).

A. R. Carr *et al.*, Three-dimensional super-resolution in eukaryotic cells using the double-helix point spread function. *Biophys. J.* **112**, 1444–1454 (2017).

V. T. Chang *et al.*, Initiation of T cell signaling by CD45 segregation at "close contacts". *Nat. Immunol.* **17**, 574–82 (2016).

S. J. Davis, S. Ikemizu, M. K. Wild, P. A. van der Merwe, CD2 and the nature of protein interactions mediating cell-cell recognition. *Immunol. Rev.* **163**, 217–36 (1998).

S. J. Davis, P. A. van der Merwe, The kinetic-segregation model: TCR triggering and beyond. *Nat. Immunol.* **7**, 803–9 (2006).

E. Edelstein, N. Amodaj, K. Hoover, R. Vale, N. Stuurman, Computer control of microscopes using μManager. *Curr. Protoc. Mol. Biol.* **14**, 1–14 (2010).

E. Hui, R. D. Vale, In vitro membrane reconstitution of the T-cell receptor proximal signaling network. *Nat. Struct. Mol. Biol.* **21**, 133–42 (2014).

J. R. James, R. D. Vale, Biophysical mechanism of T-cell receptor triggering in a reconstituted system. *Nature* **487**, 64-9 (2012).

M. D. Lew, A. von Diezmann, W. E. Moerner, Easy-DHPSF open- source software for three-dimensional localization of single molecules with precision beyond the optical diffraction limit. Available at Protocol Exchange (https://doi.org/10.1038/protex.2013.026+) (2015).

J. L. Ptacin *et al.*, A spindle-like apparatus guides bacterial chromosome segregation. *Nat. Cell Biol.* **12**, 791-8 (2010).

Y. Razvag, Y. Neve-Oz, J. Sajman, M. Reches, E. Sherman, Nanoscale kinetic segregation of TCR and CD45 in engaged microvilli facilitates early T cell activation. *Nat. Commun.* **9**, 732 (2018).

C. A. Schneider, W. S. Rasband, K. W. Eliceiri, NIH Image to ImageJ: 25 years of image analysis, *Nature Methods* **9**, 671-5 (2012).

J. Vogelsang, T. Cordes, C. Forthmann, C. Steinhauer, P. Tinnefeld, Controlling the fluorescence of ordinary oxazine dyes for single-molecule switching and superresolution microscopy. *Proc. Natl. Acad. Sci. U. S. A.* **106**, 8107–12 (2009).

D. L. Weaver, Diffusion-mediated localization on membrane surfaces. *Biophys. J.* **41,** 81-86 (1983).

L. Weimann *et al.*, Quantitative comparison of single-dye tracking analysis tools using Monte Carlo simulations. PLoS One **8**, e64287 (2013).
